# Altered Expression of Shorter p53 Family Isoforms Can Impact Melanoma Aggressiveness

**DOI:** 10.3390/cancers13205231

**Published:** 2021-10-18

**Authors:** Ana Tadijan, Francesca Precazzini, Nikolina Hanžić, Martina Radić, Nicolò Gavioli, Ignacija Vlašić, Petar Ozretić, Lia Pinto, Lidija Škreblin, Giulia Barban, Neda Slade, Yari Ciribilli

**Affiliations:** 1 Laboratory for Protein Dynamics, Division of Molecular Medicine, Ruđer Bošković Institute, 10000 Zagreb, Croatia; Ana.Tadijan@irb.hr (A.T.); nikolina.hanzic@hztm.hr (N.H.); Martina.Radic@irb.hr (M.R.); Ignacija.Vlasic@irb.hr (I.V.); lidija.petrovic0@gmail.com (L.Š.); 2 Laboratory of Molecular Cancer Genetics, Department of Cellular, Computational, and Integrative Biology (CIBIO), University of Trento, 38123 Povo, TN, Italy; f.precazzini@unitn.it (F.P.); nicolo.gavioli@iqvia.com (N.G.); lia.pinto@lih.lu (L.P.); giulia.barban@humanitas.it (G.B.); 3 Laboratory of RNA Biology and Biotechnology, Department of Cellular, Computational, and Integrative Biology (CIBIO), University of Trento, 38123 Povo, TN, Italy; 4 Laboratory for Hereditary Cancer, Division of Molecular Medicine, Ruđer Bošković Institute, 10000 Zagreb, Croatia; Petar.Ozretic@irb.hr

**Keywords:** melanoma, p53, p73, Δ160p53, isoforms, targeted therapy, resistance

## Abstract

**Simple Summary:**

*TP53* is one of the most important tumor suppressor genes, which has been found to be mutated in more than half of human cancers and is considered the “Guardian of the genome”. However, it is rarely mutated in melanoma (less than 20% of cases). Although several cancer-oriented studies focus on p53 biology, only recently have researchers started to appreciate the importance of shorter p53 isoforms as potential modifiers of p53-dependent responses. In this study, we showed that melanoma-derived cell lines express a wide array of p53 and p73 isoforms, with Δ160p53α as the most variable. For the first time, we reported that Δ160p53α, and to a lesser extent Δ160p53β, can be recruited on chromatin, and Δ160p53γ can localize in perinuclear foci; moreover, all Δ160p53 isoforms can stimulate proliferation and, potentially, migration. Lastly, we showed an increased expression of the potentially pro-oncogenic Δ40p53β isoform and a decrease in the tumor-suppressive TAp73β isoform in melanoma cells resistant to vemurafenib (BRAF inhibitor). With this study, we suggest that p53 family isoforms play a significant role in melanoma cells’ aggressiveness.

**Abstract:**

Cutaneous melanoma is the most aggressive form of skin cancer. Despite the significant advances in the management of melanoma in recent decades, it still represents a challenge for clinicians. The *TP53* gene, the guardian of the genome, which is altered in more than 50% of human cancers, is rarely mutated in melanoma. More recently, researchers started to appreciate the importance of shorter p53 isoforms as potential modifiers of the p53-dependent responses. We analyzed the expression of p53 and p73 isoforms both at the RNA and protein level in a panel of melanoma-derived cell lines with different *TP53* and *BRAF* status, in normal conditions or upon treatment with common anti-cancer DNA damaging agents or targeted therapy. Using lentiviral vectors, we also generated stable clones of H1299 p53 null cells over-expressing the less characterized isoforms Δ160p53α, Δ160p53β, and Δ160p53γ. Further, we obtained two melanoma-derived cell lines resistant to BRAF inhibitor vemurafenib. We observed that melanoma cell lines expressed a wide array of p53 and p73 isoforms, with Δ160p53α as the most variable one. We demonstrated for the first time that Δ160p53α, and to a lesser extent Δ160p53β, can be recruited on chromatin, and that Δ160p53γ can localize in perinuclear foci; moreover, all Δ160p53 isoforms can stimulate proliferation and in vitro migration. Lastly, vemurafenib-resistant melanoma cells showed an altered expression of p53 and p73 isoforms, namely an increased expression of potentially pro-oncogenic Δ40p53β and a decrease in tumor-suppressive TAp73β. We therefore propose that p53 family isoforms can play a role in melanoma cells’ aggressiveness.

## 1. Introduction

Malignant melanoma is an aggressive and invasive tumor with increasing incidence, and still without a reliable prognostic biomarker that could predict the course of the disease [1,2]. Although we are witnessing great improvements in melanoma therapy, the resistance to available therapies still persists. Therefore, it is crucial to develop novel molecular approaches that will contribute to the identification of the molecular signatures of melanoma, for a better understanding and treatment of this disease.

The tumor suppressor gene *TP53* is critically important in the cellular response to several stress signals. Activated p53 elicits cell cycle arrest, DNA repair, apoptosis, and, in some circumstances, senescence, thereby opposing tumor formation [3,4,5]. Although in metastatic melanoma the *TP53* gene is relatively rarely mutated (below 20% of cases according to the cBioPortal database for cancer genomics [6]), wild-type p53, which is present in over 80% of melanoma, does not function well as a tumor suppressor [7,8,9]. Moreover, reduced p53 levels contribute to tumor aggressiveness and resistance to therapy [10]. Overall, the role of p53 in melanoma is still disputed. Several mechanisms of p53 impairment in melanoma have been proposed, including mutations in the *CDKN2A* gene (encoding for both p16^INK4A^ and p14^ARF^, the latter of which relieves p53 from the negative regulation of MDM2 and its inactivation promotes the inhibition of p53 by MDM2) and the over-expression of MDM2, MDMX/MDM4 (an additional negative regulator of p53), or the inhibitor of apoptosis-stimulating protein of p53 (iASPP) and anti-apoptotic proteins such as BCL-2, which allow melanoma cells to become highly resistant to apoptosis [11,12]. This is particularly relevant in melanomas bearing mutant NRAS and wild-type (wt) p53 where the upregulation of MITF leads to an increase in BCL-2 protein [13]. Furthermore, recent studies have emphasized the relevance of the *TP53* gene silencing by epigenetic mechanisms, including the impact of miRNAs [12,14,15,16,17].

The p53 family consists of p53, p63, and p73 proteins. All family members share a dual gene structure due to the presence of two distinct promoters, the canonical P1, and the internal P2, from which several *TP53* mRNAs can be transcribed [18,19]. The *TP53* mRNAs can be spliced at intron 2 or intron 9; consequently, variants with different N- or C-termini can be generated. Additionally, *TP53* mRNAs can be translated starting from different codons—ATG 1, ATG 40, ATG 133, or ATG 160, resulting in p53 isoforms that differ in length [20,21]. Accordingly, the p53 isoforms can be classified as long p53 or short depending on transcription and translation initiation. The *TP53* mRNA transcripts transcribed from the P1 promoter, translated at codon 1 and/or codon 40, give rise to the long isoforms (full length, canonical p53, and Δ40p53), whereas mRNAs transcribed from the P2 can be translated at codons 133 and/or 160, encoding the short isoforms (Δ133p53 and Δ160p53) [22].

Likewise, multiple *TP73* mRNAs are generated as a result of the transcription from two promoters combined with the alternative splicing at the 5′- and 3′-ends. The transcription from the P1 promoter gives rise to a group of transcriptionally active TAp73 isoforms. Conversely, the transcription from the alternative P2 generates N-terminally truncated ΔNp73 isoforms [18]. Many parallels can be found between the functional p53, TAp73, and TAp63 on one hand, and between ΔNp73 and ΔNp63 on the other. Proteins with an intact transactivation domain (TAp73) can mimic the functions of p53 in transactivating many p53 target genes, whereas proteins without it (ΔNp73) inhibit apoptosis and show a dominant-negative effect toward p53 and TAp73 [23,24]. In addition, the Ex2/3 spliced transcript called ΔEx2/3p73 was found to be significantly upregulated in melanoma metastases [25,26]. However, the p53 isoforms cannot be categorized as oncogenic or tumor suppressive since their biological activities and their prognostic values are associated with the cell context, and the involvement of p53 isoforms in tumor formation is still being investigated.

There are twelve protein isoforms encoded by a single *TP53* gene with different protein-interacting domains and activities which can also be modified post-translationally [20,21]. This diversity leads to different subcellular localization, and, consequently, different biochemical/biological features which are also cell-type dependent. Eventually, p53-mediated cell responses are the sum of the activities of all co-expressed p53 isoforms. In the same way, *TP73*, which is essentially never mutated in cancer, encodes numerous isoforms with two main subgroups differing in N-termini: the TAp73 isoforms and the N-terminally truncated ΔNp73 isoforms. Although both groups are over-expressed in tumors, the latter ones are predominant, displaying a dominant-negative effect toward p53 and TAp73 [18]. The third member of the family, p63, was found to interact with p53 in melanoma, thereby influencing its function [27].

Most of the p53 family isoforms have the ability to tetramerize between themselves, forming heterotetramers, and to modulate the transcriptional activity. The final activity of p53 family proteins is a result of the ratio between different isoforms, and an imbalance between isoforms can favor tumor development. It is also possible that a different set of isoforms from the same gene can directly interact, potentially using the oligomerization domain. For instance, by forming heterotetramers with p53, the Δ40p53 isoform impacted melanoma cell fate, favoring apoptosis rather than cell cycle arrest [28]. Another study revealed that the Δ40p53 is expressed in melanoma cell lines and it can alter the p53-dependent responses to DNA damage [29]. We have recently studied p53 and p73 expression in a limited cohort of 32 melanoma patients and 19 matched healthy tissues, showing an increase in the expression of Δ133p53α, Δ160p53α, and ΔNp73α isoforms in tumor tissues [30]. Interestingly, we were also able to correlate higher Δ133p53β levels with a poorer prognosis [30].

Since there is great limitation in studying melanoma development given the paucity of early-stage primary tissues, in our research we employed established melanoma cell lines. We evaluated the expression of p53 and p73 isoforms both at the level of mRNA and protein in basal conditions as well as in response to DNA-damaging agents such as doxorubicin, cisplatin, etoposide, and radiotherapy. We also evaluated the effect of anti-cancer drugs used in melanoma, such as dacarbazine and, particularly, vemurafenib. Interestingly, we found Δ160p53α to be the most variable p53 isoform, and we determined its accumulation in chromatin-enriched cell fractions and an augmented proliferation and migration of H1299 cells stably over-expressing Δ160p53α, β, or γ. Lastly, we generated two different vemurafenib-resistant melanoma cell lines and we demonstrated only subtle changes in p53 isoform expression, specifically a slight but significant alteration of Δ133p53α and β, and Δ40p53β level. Meanwhile, the expression of p73 isoforms was differently modulated in the two resistant sublines. While in primary melanoma cells the expression of both TAp73 and ΔNp73 isoforms, as well as the melanoma-relevant isoform ΔEx2/3p73, was reduced, in metastatic cells the expression of all tested isoforms was increased compared to the control parental cells.

## 2. Materials and Methods

### 2.1. Cell Lines and Culture Conditions

A total of 27 melanoma-derived cell lines were used in this study; 6 were obtained from primary melanomas while 21 were isolated from metastatic sites (see Table S1 for details). SK-MEL-5, SK-MEL-28, WM-266, and G-361 cells were received from Dr. Alessandra Bisio (CIBIO Department, University of Trento, Povo (TN), Italy) [31]; A375, MeWo, RPMI-7951, SK-Mel-24, and SK-Mel-3 from Dr. Andreja Ambriović Ristov (Ruđer Bošković Institute, Zagreb, Croatia); while Ma-Mel-8a, -35, -54a, -55, -61b, -61f, -86a, and -86c cell lines were obtained from Dr. Annette Paschen (Department of Dermatology, University Hospital Essen, Essen, Germany). Interestingly, Ma-Mel-61b, -61f, and Ma-Mel-86a, -86c cells originate from 2 melanoma cases (61 and 86, respectively) where 2 metastases for each case were isolated and displayed different sensitivity to the BRAF inhibitor vemurafenib [32,33]. All the other melanoma-derived cell lines were obtained from Dr. Daniele Bergamaschi (Centre for Cell Biology and Cutaneous Research, Blizard Institute, Barts and The London School of Medicine and Dentistry, London, UK). NIH-H1299 cells used for transient and stable transfection experiments were received from Dr. Daniel Menendez (National Institute for Environmental Health Sciences, NIHES, NIH, Research Triangle Park, NC, USA). HEK293T packaging cells were obtained from Prof. Jürgen Borlak (Hannover Medical School, Hannover, Germany). Cells were grown in Dulbecco’s modified Eagle medium (DMEM), Eagle’s minimum essential medium (αMEM) (both Sigma-Aldrich/Merck, Darmstadt, Germany) or RPMI 1640 (Lonza, Basel, Switzerland), supplemented with 10% or 15% fetal bovine serum (Thermo Fisher Scientific, Waltham, MA, USA), 1% streptomycin–penicillin (Sigma-Aldrich/Merck), 1 mM sodium pyruvate (Life Technologies, Carlsbad, CA, USA) and 2 mM L-glutamine (Sigma-Aldrich/Merck). Primary Human Melanocytes (HEMa, Thermo Fisher Scientific) were maintained in Medium 254 with the addition of Human Melanocyte Growth Supplement (HMGS-2, all Thermo Fisher Scientific). Cells were maintained in a humidified incubator at 37°C under 5% CO_2_ atmosphere. All cell lines were tested free of mycoplasma. All the commercially available cell lines obtained from collaborators were purchased by them from different cell banks, and we received them at very low passage number from the original clones.

### 2.2. RNA Extraction, cDNA Synthesis, and Quantitative PCR

RNA was extracted from cell lines using the RNeasy (Qiagen, Hilden, Germany) or PureLink RNA Mini (Thermo Fisher Scientific) kits following the manufacturer’s recommendations. Genomic DNA was removed by enzymatic digestion with the DNase I either directly on the column or in vitro (ThermoFisher Scientific) prior to cDNA synthesis. One or two micrograms of RNA was then converted into cDNA using the RevertAid First Strand cDNA Synthesis (ThermoFisher Scientific) or High-Capacity cDNA Reverse Transcription (Applied Biosystems, Waltham, MA, USA) kit following the manufacturer’s instructions. To distinguish the 9 different *TP53* isoforms, a quantitative PCR was performed according to the recently developed method we adopted and slightly modified, as previously described [30]. A total of 25 ng of cDNA was used for pre-amplifications using Go-Taq (Promega, Madison, WI, USA). qPCR was performed with 1:400 (for full length and Δ40p53) or 1:200 (for Δ133p53) diluted pre-amp PCRs using the qPCRBIO Sybr Green Mix (PCR Biosystems, Resnova, Rome, Italy) or Takyon Low Rox SYBR MasterMix dTTP Blue (Eurogentec, Liège, Belgium) and quantified with the CFX384 and CFX96 Real-Time PCR Detection Systems (Bio-Rad Laboratories, Hercules, CA, USA) instruments (40 cycles of 15 s 95 °C, 20 s 63 °C, and 10 s 72 °C, with the final step of 10 s 72 °C). Results were analyzed with CFX Manager Software v3.1 (Bio-Rad Laboratories), normalized with Ct values for total p53, and antilog values of 2^−ΔCt^ were presented as bars using GraphPad Prism 8 (GraphPad Software, San Diego, CA, USA) or as a heatmap using Excel 2010 (Microsoft Office, Redmond, WA, USA). Primers were validated for quality and efficiency, positions are presented in Figure S1, and the sequences were reported previously [30] (Eurofins Genomics, Ebersberg, Germany). qPCR analysis of ΔNp73 and TAp73 expression was performed on 100 ng cDNA using TaqMan Gene Expression Master Mix (Applied Biosystems) and TaqMan Gene Expression Assays (Thermo Fisher Scientific) on the StepOnePlus Real-Time PCR System (Applied Biosystems). Thermal cycling conditions were as follows: 2 min 50 °C and 10 min 95 °C, followed by 40 cycles of 15 s 95 °C and 1 min 60 °C. Gene expression assays used: Hs01065727_m1 for ΔNp73, Hs00232088_m1 for TAp73, Hs00939627_m1 for glucuronidase beta (GUSB), and Hs00427620_m1 for TATA-Box Binding Protein (TBP). Signals were analyzed with StepOnePlus Software v2.3 (Applied Biosystems), normalized with GeoMean of Ct values for GUSB and TBP and presented as antilog values of 2^−ΔCt^. qPCR analysis of ΔEx2/3p73 expression was performed on 25 ng cDNA using primers: F: 5′-TGCAGGCCAGTTCAATCTGC-3′, R: 5′-TCGGTGTTGGAGGGGATGACA-3′ and Luna Universal qPCR Master Mix (New England Biolabs, Ipswich, MA, USA) on a CFX96 Real-Time PCR Detection System (Bio-Rad Laboratories) instrument. Thermal cycling conditions were as follows: 1 min 95 °C, followed by 40 cycles of 15 s 95 °C and 30 s 59 °C. Results were analyzed with CFX Manager Software v3.1 (Bio-Rad Laboratories), calibrated with the geometric mean of Ct values for GUSB and TBP, and antilog values of 2^−ΔCt^ were presented as bars using GraphPad Prism 8 (GraphPad Software, San Diego, CA, USA).

### 2.3. Transient Transfections

The complementary DNA to *TP53* isoform RNA (kindly provided by Dr. J.C. Bourdon, University of Dundee, Dundee, UK) was cloned into a pcDNA3-expressing vector. H1299 cells were seeded into 6-well plates 24 h prior to transfection in order to reach an appropriate confluence (70–80%). Plus Reagent (Life Technologies, Carlsbad, CA, USA) or Turbofect (Thermo Fisher Scientific, Waltham, MA, USA) and p53 isoform expression plasmids (500 ng) were diluted in 100 µL of Opti-MEM medium (Life Technologies) with a ratio 1:1 (µL of Plus reagent: µg of transfected DNA). Lipofectamine LTX Reagent (3:1) (Life Technologies) was diluted in 100 µL of Opti-MEM medium. Formed complexes were then added to plated cells and proteins were harvested 24 h later. For knockdown experiments, non-specific siRNA control scrambled or p53 isoform-specific small interfering RNA (siRNA) duplexes were synthesized at Eurogentec. The siRNA sequences used were as follows: si(-) (non-specific); p53α siRNA 5′-GUGAGCGCUUCGAGAUGUU-3′; p53β siRNA 5′-GGACCAGACCAGCUUUCAA-3′; p53γ siRNA 5′-CCCUUCAGAUGCUACUUGA-3′; pan-p53 (targets all isoforms) 5′-GACUCCAGUGGUAAUCUAC-3′. Reverse transfection was performed using Lipofectamine RNAiMAX Reagent (Thermo Fisher Scientific) following the manufacturer’s instructions. Cells were harvested 48 h after transfection.

### 2.4. FASAY Assay

To determine the presence of *TP53* mutations in melanoma cell lines with unknown or uncertain *TP53* status, we performed the FASAY (Functional Analysis of Separated Alleles in Yeast) assay as previously described [34,35]. Briefly, the p53 coding sequence was amplified with specific primers (p53-P3: 5′-ATTTGATGCTGTCCCCGGACGATATTGAAC-3′ and p53-P4: 5′- ACCCTTTTTGGACTTCAGGTGGCTGGAGTG-3′) from the cell of interest cDNA, and co-transformed with the LiAc method [36] into the yeast strain yIG397, along with the linearized plasmid pRDI22, which contains the selectable marker LEU2. Transformant yeast colonies presenting a wild-type p53 gave rise to white colonies, while cells with a non-functional mutant p53 originated red colonies on the same selective plates. The percentage of red colonies was used to verify the *TP53* status: below 20% = homozygous wild-type *TP53*, >50% = heterozygous, and 100% homozygous mutant *TP53*. The specific *TP53* mutation was confirmed by direct sequencing on purified p53-specific PCR products using yeast colony PCR from three independent red colonies amplified with p53-P3 and p53-P4 primers.

### 2.5. Plasmids’ Subcloning

To obtain H1299 cell lines stably over-expressing Δ160p53α, β, and γ isoforms, lentiviral vector-mediated transduction of these genes was selected as a good and efficient strategy. The inserts containing the cDNA of Δ160p53β and Δ160p53γ isoforms were taken from a pCDNA3.1(+) plasmid (obtained from Dr. Jean-Christophe Bourdon, University of Dundee, Dundee, UK), whereas Δ160p53α insert was obtained from a pRS414 plasmid (a vector for expression in yeast obtained from Dr. Cecile Voisset, Brest, France), and transferred to pAIP, the recipient plasmid needed for the lentiviral vector production. For both procedures, we used double digestion with BamHI-HF and EcoRI-HF endonucleases (New England Biolabs) followed by dephosphorylation of the backbone with Antarctic phosphatase (New England Biolabs). Subsequently, the ligation of purified fragments (inserts extracted from agarose gels with QIAquick spin columns—Qiagen) was performed with T4 ligase (New England Biolabs). Positive *E. coli* Stbl3 colonies containing the desired expression vectors were screened and checked by direct Sanger sequencing, and transfection-grade (Endo-free) plasmids pAIP-empty, -Δ160p53α, -Δ160p53β, and -Δ160p53γ were obtained using the PureYield Plasmid Midiprep System (Promega, Madison, WI, USA).

### 2.6. Lentiviral Vectors Production

To produce lentiviral vectors, HEK293T cells were used as packaging cells. A total amount of 3 × 10^6^ HEK293T cells were seeded into a 10 cm dish for each transfection and were left to grow in DMEM growth medium overnight at 37 °C with 5% CO_2_. The day after, the culture medium was changed with 5 mL OptiMEM. Cells were then transfected using 2 × PEI solution (Sigma-Aldrich/Merck) with each pAIP-derived plasmid (10 µg) mixed with pCMV-VSVg (3 µg) and with pCMV-dR8.9 (7 µg) plasmids and incubated at 37 °C for 16 h when the medium was changed with standard DMEM. After 48 h, lentiviral vectors were harvested from the exhausted medium. The supernatant containing lentiviral particles was carefully collected after centrifugation at 500 g for 5 min and filtered through 0.45 μm filters. Lentiviral vector titration was performed by PERT (Product-Enhanced Reverse Transcriptase assay) with the help of Prof. Massimo Pizzato (CIBIO Department, University of Trento, Povo (TN), Italy) to quantify the amount of lentiviral vector produced by HEK293T cells [37].

### 2.7. H1299 Transduction and Generation of Stable Clones Expressing Δ160p53 Isoforms

A total amount of 30,000 H1299 cells/well were seeded into a 24-well plate and left growing in RPMI complete medium at 37 °C overnight. The day after RPMI medium was discharged, 1 RTU (Reverse Transcriptase Units)/well of lentiviral-vector-containing medium was added to the cells. After incubation for 72 h, cells were transferred into 6-well plates and positive clones were selected by adding 1.5 μM puromycin (Life Technologies) in RPMI complete medium, and the surviving ones expanded. Single clones over-expressing Δ160p53α, Δ160p53β, and Δ160p53γ (or the empty vector) were obtained through serial dilutions into 96-well plates and cultured in RPMI complete medium with 1 μM puromycin.

### 2.8. Treatments

The A375M and WM793B cells were treated with various doses of γ-irradiation (from 5 to 30 Gy, at the average dose rate 2 Gy/min) using ^60^Co source at the Ruđer Bošković Institute in Zagreb, Croatia or with cisplatin (Sigma-Aldrich/Merck) or etoposide (Sandoz, Holzkirchen, Germany) (both IC50 of 1 μM, defined with MTT assay). Alternatively, doxorubicin (0.75–1.5 μM, Sigma-Aldrich/Merck) and dacarbazine (5-10 μM, Sigma-Aldrich/Merck) were used to treat most of the cell lines. Cells were harvested for protein analysis at different time points and protein extracts were analyzed by Western blotting. In addition, A375M and WM793B were treated for 24 h with different doses of vemurafenib (1 µM, 2 µM, or 5 µM) where DMSO treatment was used as a negative control.

### 2.9. Protein Extraction and Western Blotting

Protein extraction from cellular pellets was performed with NP-40 lysis buffer (50 mM TrisHCl pH 8, 150 mM NaCl, 10% Glycerol, 1% Nonidet P-40) supplemented with protease inhibitors cocktail (Complete, Mini Protease Inhibitor Cocktail tablets, Roche, Basel, Switzerland) or direct lysis with Promega passive lysis buffer (Promega) and by sonication for 30 s at 25% amplitude (Sonics VibraCell Processor, Newtown, CT, USA) or UP50H-Compact Lab Homogenizer (Hielscher Ultrasonics, Teltow, Germany). Protein extracts were quantified with BCA Protein Assay (Pierce, ThermoFisher Scientific), measuring the absorbance at 562 nm with the Infinite M200 (Tecan, Männedorf, Switzerland), or at 570 nm with the Multiskan MS (Labsystems, Vantaa, Finland) multi-plate reader. For detecting p53 and p73 isoforms, regular or three-stage (for KJC8 and KJCγ antibodies) Western blot was performed. Proteins (40–50 μg) were prepared in 1 × Laemmli sample buffer (50 mM Tris-HCl pH 6.8; 100 mM DTT; 2% SDS; 10% glycerol; 0.0025% bromphenol blue), denatured by heating for 5 min at 95 °C, separated on 9–12% SDS–polyacrylamide gels, and transferred to nitrocellulose membranes (Merck Millipore). Mouse pantropic anti-p53 antibody (clone DO11, 1:1000), sheep pantropic anti-p53 antibodies (clones SAPU and KJC12, 1:5000), mouse anti-p53α isoform antibodies (clones 421 and BP50.10, 1:1000), rabbit and sheep anti-p53β isoform antibodies (clones KJC8 and β-sheep, 1:1000 and 1:5000, respectively), and rabbit anti-p53γ isoform antibody (clone KJCAγ, 1:1000) were all kindly provided by J.C. Bourdon. Regarding other antibodies, mouse monoclonal anti-p53 (clone DO1; Santa Cruz Biotechnology, Dallas, TX, USA; 1:2000), mouse monoclonal anti-β-actin (60008-1-1g, Proteintech, Rosemont, IL, USA; 1:3000), secondary anti-mouse (HRP conjugated anti-IgG; Cell Signaling Technology, Danvers, MA, USA; 1:3000), secondary anti-rabbit (IgG HRP-linked antibody; Cell Signaling, USA; 1:3000), secondary anti-rabbit (AffiniPure goat anti-rabbit, unconjugated; Jackson ImmunoResearch Europe, Ely, UK; 1:1000), secondary anti-sheep (peroxidase-conjugated AffiniPure donkey anti-sheep IgG; Jackson ImmunoResearch Europe; 1:10,000), and secondary anti-goat (AffiniPure donkey anti-goat IgG HRP-conjugated; Jackson ImmunoResearch Europe; 1:5000) were used. p53 and p73 isoforms were visualized using SuperSignal Western Blot Substrate Pico and Femto (3:1) and the β-actin with Western Lightning Chemiluminescence Reagent Plus (PerkinElmer, Waltham, MA, USA), using the Alliance 4.7 imaging system or Alliance Q9 mini (both UVitec, Cambridge, UK). Alternatively, immune-reactive bands were visualized using the ECL Select reagent (Amersham, GE Healthcare, Chicago, IL, USA) and detected using the ChemiDoc XRS+ (Bio-Rad Laboratories) or the Alliance LD2 (UVitec) documentation systems, as previously described [38]. The expression of proteins of interest was quantified using Image Lab software 6 (Biorad, Hercules, CA, USA) and normalized to the expression level of β-actin or to intensity of the naphthol blue membrane staining of the same samples. Naphthol blue membrane staining was performed immediately after transfer by incubating the membrane for 1 min on shaker in a naphthol blue staining solution (10% (*v*/*v*) methanol, 2% (*v*/*v*) acetic acid, 0.1% (*w*/*v*) naphthol blue), after which the membrane was incubated in the destaining solution (50% (*v*/*v*) methanol, 7% (*v*/*v*) acetic acid) for 15 min on a shaker and then photographed. The sum of the signals from three random strips of naphthol-blue-stained membrane (a representative one is shown in the Figures 3C,D, and 6B) was used for normalization of the expression of a protein of interest. All original uncropped western blot can be found in Figure S7.

### 2.10. Cytoplasmic–Nuclear Fractionation

In order to collect protein fractions from the cytoplasm, the nucleoplasm, and the chromatin, we used an already established micrococcal nuclease-based (MNase) protocol we recently adopted [39,40,41]. Briefly, cellular pellets were lysed by incubation in ice with 250 μL of NSB (Nucleus Separation Buffer—10 mM Hepes, 10 mM KCl, 1.5 mM MgCl_2_, 0.34 M sucrose, 10% glycerol, 1mM DTT, 0.1% Triton X-100—supplemented with protease inhibitor cocktail). After centrifugation at 1300 rpm at 4 °C for 10 min, the supernatant, which contains a cytosolic fraction, was collected. Nuclei were then resuspended in 100 μL of NSB supplemented with 1 mM CaCl_2_ and 2000 gel units/mL MNase (New England Biolabs) and kept at 37 °C for 10 min. The reaction was blocked by the addition of 2 mM EGTA (Sigma-Aldrich/Merck), and nuclear-soluble fractions were collected as supernatants after centrifugation at 1300 rpm at 4 °C for 10 min. Samples were then resuspended in 100 μL of NSB, with 600 mM NaCl, and kept rotating at 4 °C overnight. The samples were then centrifuged at 1300 rpm at 4 °C for 10 min and the supernatants containing the chromatin-enriched fractions were collected. Protein extracts from each fraction were loaded volumetrically into 10% acrylamide gels as follows: 15 μL and 10 μL per sample for cytoplasmic and chromatin-enriched fractions, respectively. Western blot analysis was performed as described above.

### 2.11. Immunofluorescence

Firstly, in each well of a 12-well plate, a thin glass coverslip was placed, then a total number of 80,000 H1299 cells, transfected and selected as previously described, were seeded and grown in RPMI + 1 μM puromycin overnight. The day after, the culture medium was discarded and cells were washed 3 times with 1 × PBS and fixed with 800 μL of a 4% formaldehyde (Sigma-Aldrich/Merck) solution at room temperature for 20 min. Fixation was blocked by adding 800 μL of 0.1M glycine and kept at room temperature for 5 min. The glycine solution was then discarded, cells were washed 3 times with 1 × PBS, and cells were treated with 800 μL of a 1% BSA + 0.2% Triton X-100 in PBS solution at room temperature for 10–15 min to permeabilize the cellular membranes. The cell-covered coverslips were placed with the cell layer facing downwards, on a small drop of about 30 μL of a primary antibody solution (either DO-12 or SAPU antibodies diluted 1:200 in PBS), and placed on a parafilm layer inside a humidified chamber for 1 h at room temperature. The coverslips were washed 3 times with 1X PBS and, again, the cell-covered coverslips were placed downwards on a 30 μL drop of secondary antibody solution (anti-mouse (Life Technologies) or anti-sheep (Jackson ImmunoResearch Europe) AlexaFluor 488 conjugated antibodies diluted 1:100 in PBS) in the same humidified chamber and were left, away from light, at room temperature for 1 h. The coverslips were then washed 3 times with 3 × PBS and the cell-covered coverslips were placed downwards on a 5 μL drop of DAPI solution (diluted 1:10,000 in PBS to counterstain the nuclei) in the same humidified chamber and were left, away from light, at room temperature for no more than 5 min. The coverslips were finally taken from the humidified chamber, washed 3 times with 3 × PBS, and mounted on a microscope slide with the use of a drop of FluorSave mounting medium (Merck Millipore). The slides were left to dry at room temperature overnight. Images were acquired at the CIBIO Advanced Imaging Facility by Zeiss Observer provided with an Apotome module (Zeiss, Oberkochen, Germany) and 63 × oil immersion objective, and were analyzed with the use of Zen 2.5 pro software (Zeiss).

### 2.12. Colony Formation Assay

As a proliferation assay, we performed a colony formation assay with H1299 cells over-expressing Δ160p53α, Δ160p53β, and Δ160p53γ. A total of 1000 cells were seeded into 6-well plates and grown for two weeks, changing the growth medium twice per week. Visible clones were then washed in 1 × PBS and stained with 0.1% crystal violet and 20% methanol solution for 20 min. Colonies were then washed thoroughly in 1 × PBS and counted manually.

### 2.13. MTT Assay

The MTT assay was used to test the sensitivity of the cells to vemurafenib or etoposide, and was performed as previously described [42]. Briefly, cells were seeded into 96-well plates and, after 24 h, cells were treated with the indicated doses. After 72 h of treatment, MTT reagent solution (Sigma-Aldrich/Merck) was added into each well and the plate was kept at 37 °C for 3 h. Then, formazan crystals were solubilized with the addition of DMSO, and absorbance was measured at 570 nm with the Infinite M200 (Tecan, Männedorf, Switzerland) or the Multiskan MS (Labsystems) multi-plate readers.

### 2.14. Generation of Vemurafenib-Resistant Melanoma Cell Lines

Two vemurafenib-resistant melanoma cell lines—A375M-R and WM793B-R (both *BRAF* V600E/*TP53* wt)—were generated. A375M cells were initially treated with 0.5 μM vemurafenib (PLX-4032, Selleckchem, Houston, TX, USA) for 2 weeks followed by 6 weeks’ treatment with increasing concentrations: 0.75 μM, 0.8 μM, 0.9 μM, and 1 μM. WM793B cells were treated with 3 µM vemurafenib for 2 weeks followed by 4 µM for 6 weeks. After 2 months of treatment, IC50 was tested using MTT assay and compared to the IC50 of parental cells. R1 sublines were grown in vemurafenib-supplemented medium for 2–7 months, and R2 sublines for 7–12 months. In addition, A375M and WM793B cells were treated for 24 h with different doses of vemurafenib (1 µM, 2 µM, or 5 µM) where DMSO treatment was used as a negative control.

### 2.15. Wound-Healing Assays

The scratch or wound-healing assay was performed using H1299 cells over-expressing Δ160p53α, Δ160p53β, and Δ160p53γ. A total of 2.5 × 10^5^ cells were seeded into 6-well plates and grown to reach 100% confluence. Scratches were generated using 10 µl micropipette tips, and the growth medium was changed to remove detached cells. Images were taken at 0 h, 8 h, and 24 h time points using Leica DMIL LED light microscope (Leica Microsystems, Wetzlar, Germany). Migration was then quantified at 0 h and 24 h using the Wimasis Image Analysis tool (Onimagin Technologies SCA, Córdoba, Spain).

### 2.16. Statistical Analysis

Since the normal distribution of continuous variables was confirmed using the D’Agostino–Pearson test, the parametric statistical test was used for gene expression. To determine if there was a statistically significant difference in the expression between subgroups, one-way Anova with a post hoc Tukey–Kramer test was used. Statistical analyses were performed using MedCalc, version 18.11.3 (MedCalc Software, Ostende, Belgium) and GraphPad Prism 8 (GraphPad Software, San Diego, CA, USA).

## 3. Results

### 3.1. Different p53 and p73 Isoforms Are Expressed in Melanoma Cell Lines

**Figure 1:**
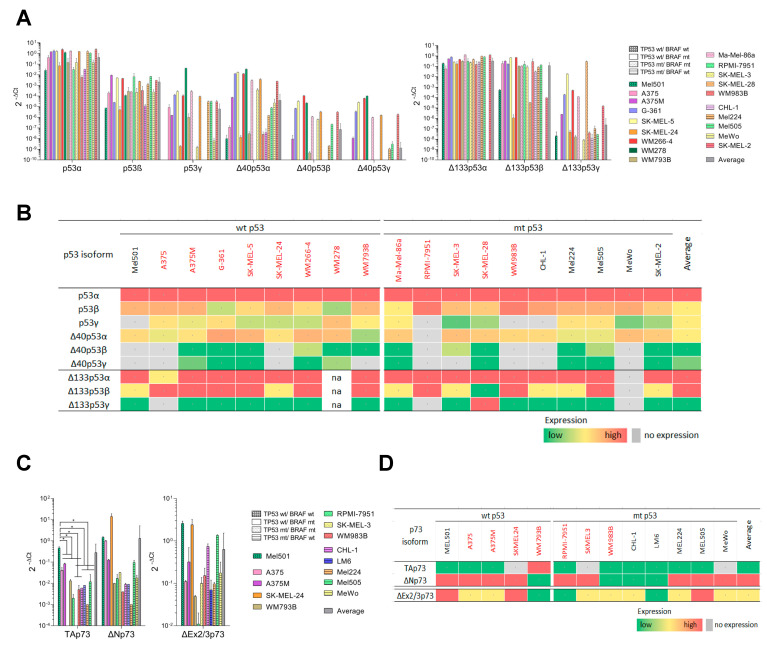
The gene expression of p53/p73 isoforms in melanoma cell lines. (**A**) Expression of the *TP53* gene in the melanoma cell lines. The bars show relative expression levels of *TP53* isoforms analyzed by pre-amplification followed by qPCR, normalized to the expression level of total *TP53*. The cell lines are grouped according to their p53/BRAF mutational status. (**B**) Heatmap display of relative *TP53* isoforms’ expression relative to the values of all isoforms (corresponding to the panel 1A) in each cell line; green color indicates the lowest, while red color the highest gene expression. Grey color indicates lack of expression. Isoform names written in black indicate BRAF wt, and in red, BRAF mt. (**C**) Expression of the *TP73* gene in the melanoma cell lines. Relative expression levels of *TP73* isoforms were analyzed by qPCR. The expression was normalized to the geometric mean of GUSB and TBP. Results are presented on a negative log scale. (**D**) Heatmap display of relative ΔNp73 and TAp73 expression (corresponding to the panel 1C left) in each cell line, and display of relative ΔEx2/3p73 expression (corresponding to the panel 1C right); green color indicates the lowest, while red color the highest gene expression. Grey color indicates lack of expression. Isoform names written in black indicate BRAF wt, and in red, BRAF mt. Presented are the averages of two independently performed experiments ± SD. Two-way ANOVA with Tukey’s multiple comparison test was used to determine differences in gene expression between groups based on mutation status. * = *p* < 0.05.

Gene expression analysis of p53 and p73 isoforms was performed on 19 and 13 melanoma cell lines, respectively. The *TP53* isoforms were pre-amplified in two separate pre-amplification PCR reactions, giving a “long” and “short” template for quantitative real-time PCR reactions and analysis. The primers were designed to detect 12 isoforms of p53 individually (Figure 1A,B). TaqMan probes were used to detect all splice variants of TAp73 and ∆Np73, and the SYBR Green approach for ∆Ex2/3p73 (Figure 1C,D). qPCR analysis failed to detect the expression of any p53/p73 isoform in primary melanocytes, but most of the isoforms were expressed in melanoma cell lines. A high expression of p53α and ∆133p53α was demonstrated, followed by p53β, ∆40p53α, and ∆133p53β (Figure 1A,B). No correlation was observed between the expression of any isoform with p53 mutation status or BRAF V600E mutation, or between primary and metastatic cell lines.

We were able to determine the gene expression of three cancer-relevant *TP73* isoforms, TAp73, ∆Np73, and ΔEx2/3p73, the latter of which was found to be upregulated in metastatic melanoma [25,26] (Figure 1C,D). We found that the expression of ∆Np73 was higher than full-length (FL) isoform TAp73. Interestingly, the expression of TAp73 was significantly higher in the BRAF wt/p53 wt cell line Mel501 than all of the other mutation groups. No difference was found in p73 isoform expression between primary and metastatic cell lines.

**Figure 2:**
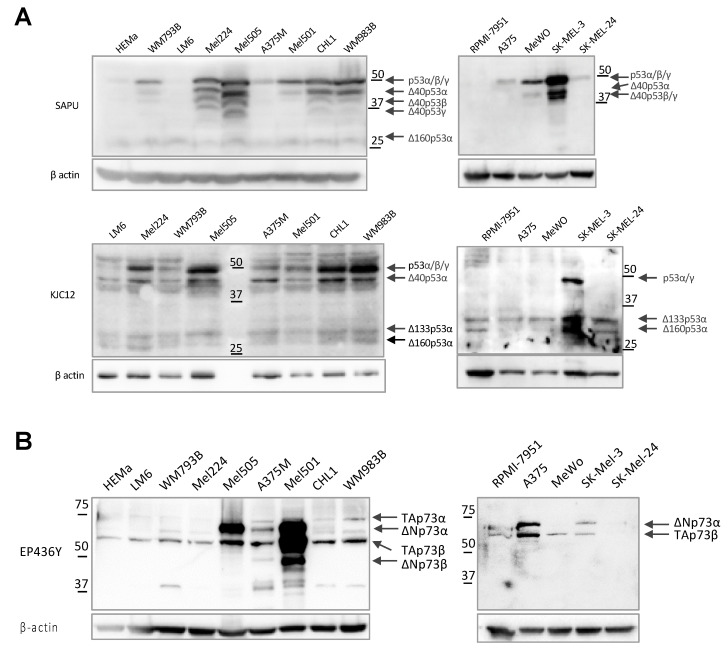
The protein expression of p53/p73 isoforms in melanoma cell lines. (**A**) Western blot analysis of endogenous p53 expression in melanoma cell lines. Proteins from melanocytes (HEMa) and melanoma cell lines expressing wt p53 (WM793B, A375M, Mel501, A375, SK-Mel-24) or mut p53 (Mel-224, Mel-505, CHL1, WM983B, MeWo, SK-Mel-3, RPMI-7951), or that were devoid of p53 expression (LM6, [45]), were extracted as described. Fifty micrograms of protein extracts were analyzed by Western blot and p53 expression was revealed using either sheep polyclonal SAPU or KJC12 anti-human p53 antibodies. The anti-actin antibody was used as the loading control. (**B**) Western blot analysis of endogenous p73 expression in melanoma cell lines. Proteins from melanocytes (HEMa) and melanoma cell lines expressing wt (WM793B, A375M, Mel501, A375, SK-Mel-24) or mut p53 (Mel-224, Mel-505, CHL1, WM983B, MeWo, SK-Mel-3, RPMI-7951), or that were devoid of p53 expression (LM6), were extracted as described. Fifty micrograms of protein extracts were analyzed by Western blot. p73 expression was revealed using rabbit monoclonal EP436Y anti-p73 antibody. The anti-actin antibody was used as the loading control. Representative data of three independent experiments yielding similar results are shown.

Protein expression analysis of p53 and p73 isoforms was performed in primary melanocytes and 13 melanoma cell lines. The expression of six p53 isoforms differing by both N- and C-termini (p53α, p53β, ∆40p53α, ∆133p53α, ∆133p53β, and ∆160p53α) (Figure 2A) and four p73 isoforms (TAp73α, TAp73β, ∆Np73α, and ∆Np73β) was determined by Western blot analysis (Figure 2B). The obtained data confirm the diverse expression of p53/p73 isoforms in melanoma cell lines. As expected, cells harboring a mutated form of p53 showed a stronger basal signal for p53α, given it is not constitutively degraded by MDM2 (as visible for CHL1, WM983B, MeWo, and SK-MEL-3) [43,44].

However, Mel224 and Mel505 cells, which were previously reported to express a wt p53 protein but with an impaired pathway [27], resulted in a very strong basal level of p53α, a classical sign of the presence of a mutant form of p53. Therefore, we used the yeast functional assay (FASAY) to verify their *TP53* status. As shown in Figure S2, transformant yeast cells showed a 100% red phenotype, a typical characteristic of a mutation in both *TP53* alleles or in the case of LOH with loss of the wild-type allele. To determine the nature of the *TP53* mutations, three red colonies from both samples were PCR amplified and sequenced. The results demonstrate the presence of a single *TP53* mutation in the Mel224 cell line (H179Y), a loss of function mutation, and a double *TP53* mutation in the Mel505 cell line (R273H; P309S), R273H being one of the hotspot *TP53* complete loss of function mutations more commonly found in human cancers. These results confirm the hypothesis of Mel224 and Mel505 cells both bearing a mutant form of p53.

### 3.2. Common Anti-Cancer Therapies Influence the Expression of p53 and p73 Isoforms

**Figure 3:**
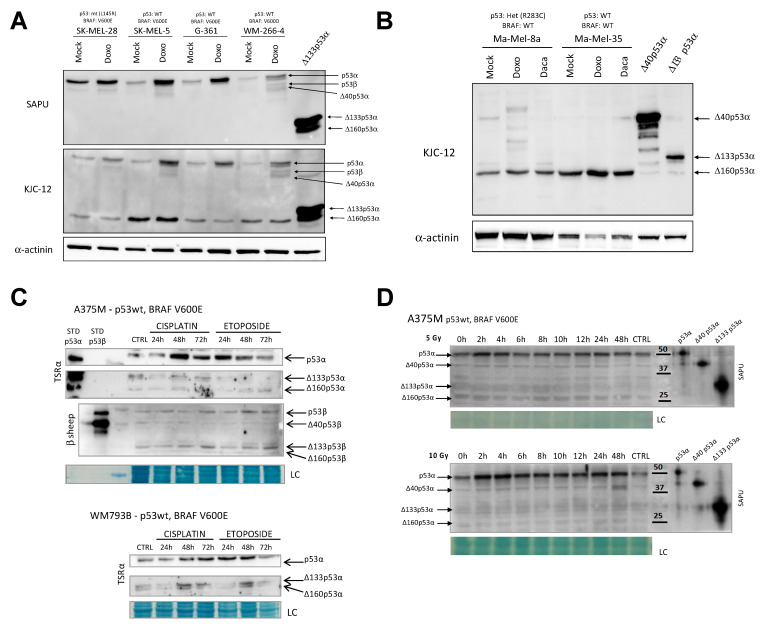
The expression of endogenous p53 isoforms in response to DNA-damaging agents commonly used in the clinic to treat cancer patients was evaluated in melanoma cell lines. Cells were treated with 1.5 µM doxorubicin (**A**), 10 µM dacarbazine (**B**), or 1 µM cisplatin or etoposide (**C**), and (**D**) γ-irradiated with 5 and 10 Gy (CTRL, non-irradiated cells; 0 h, cells collected immediately upon irradiation). Cells were harvested 24 h after the treatment (**A**,**B**), or at different time points after the treatment as indicated (**C**,**D**). α-actinin (**A**,**B**) and naphthol blue (**C**,**D**) were used as loading controls. To better identify the different p53 isoforms, transient transfections in the p53-null H1299 cells with single isoforms were included (arrows indicate the position on the blot of the intended p53 isoform, given that p53 isoforms can be post-translationally modified and multiple isoforms can be produced starting from downstream ATGs). (**A**,**B**) Respectively, 50 µg for the endogenous expression or 12.5 µg for the over-expression of p53 isoforms were loaded into the gels. (**C**,**D**) A total of 50 μg of proteins was analyzed by Western blot. Representative data of three independent experiments yielding similar results are shown.

In order to reveal the expression pattern of a specific isoform in response to DNA damage, we treated melanoma cells with doxorubicin (Figure 3A,B; Figures S3 and S6), dacarbazine (Figure 3B; Figures S3 and S6), or cisplatin and etoposide (Figure 3C), or exposed them to γ-irradiation (Figure 3D). As expected, doxorubicin stimulated the stabilization of FL p53α in all wt p53 cellular systems (more visible using SAPU antibody) (Figure 3A,B; Figures S3B and S6A–C,F–G). Additionally, in a few of the cells expressing a mutant version of p53, FL p53α was stabilized (Figure 3A; Figure S3A–C). Interestingly, among the large panel of tested cell lines, an isoform with low molecular weight was clearly the most variable. In order to determine if this isoform was Δ160p53α or Δ133p53β, we repeated the experiment using isoform-specific antibodies. As shown in S3 S3B–D, we demonstrated that this variable p53 isoform was Δ160p53α. Indeed, we were able to distinguish cells with lower, medium, or high endogenous expression levels of Δ160p53α, particularly elevated in SK-Mel-5 and Mel-224 cells. Noteworthy is that in Ma-Mel-35, the treatment with doxorubicin induced the expression of Δ160p53α (Figure 3B). When melanoma cell lines A375M and WM793B (both harboring wt p53) were treated with cisplatin and etoposide, we observed stabilization of FL p53α, except after 72 h of treatment with etoposide. Using α- and β-specific anti-p53 antibodies, we were able to distinguish the expression of α and β isoforms. The treatment with cisplatin and etoposide induced the expression of all detected isoforms, including Δ160p53α, as well as Δ160p53β, specifically in A375M cells after 72 h of treatment (Figure 3C; Figure S3G).

After γ-irradiation of A375M and WM793B (both wt p53, BRAF V600E) cell lines, the expression of FL p53α was higher than in untreated cells or in cells harvested immediately upon irradiation (0 h), (Figure 3D; Figure S3E,G). Additionally, we observed slightly higher levels of Δ40p53α, Δ133p53α, and Δ160p53α in A375M cells after the treatment compared to control, untreated cells, particularly when irradiated with a lower dose, 5 Gy (Figure 3D; Figure S3G). Furthermore, the highest expression of both TAp73β and ΔNp73α isoforms was detected 2 and 4 h after irradiation, with ΔNp73α being more strongly expressed (Figure S3F). In the Mel505 cell line, harboring a p53 mutation, we used α- and β-specific anti-p53 antibodies to distinguish different expression patterns of α and β isoforms after γ-irradiation. The expression of all p53 isoforms was induced upon the treatment (Figure S3E). Moreover, we detected TAp73β, ΔNp73α, and ΔNp73β isoforms, which all were induced by the treatment, with ΔNp73α being the most pronounced (Figure S3F).

### 3.3. Δ160p53 Isoforms Can Play a Relevant Role in Cancer Cell Aggressiveness

Since Δ160p53 was the most variable p53 isoform in expression among the different melanoma-derived cell lines, even in untreated condition, and based on the limited information available in the literature, we decided to generate stable clones over-expressing Δ160p53 isoforms in H1299 p53-null cells. To verify the newly generated stable clones, Western blot was performed using SAPU antibody. As expected, H1299 cells expressed the desired Δ160p53 isoforms, which migrated comparably with the same p53 isoforms transiently transfected in H1299 cells (Figure S4A). To verify the specificity of the visualized bands, α- (BP53-10) and β-specific (KJC-8) antibodies were used, which confirmed the nature of the detected isoforms (Figure S4B). Surprisingly, even though the total amount of protein extracts of transfected H1299-derived samples was the same, Δ160p53γ seemed to be expressed at lower levels (Figure S4A). Due to the intrinsic distribution of its epitopes, it is well known that SAPU antibody detects preferentially longer p53 and/or α-specific isoforms [20]. This observation can explain the apparent stronger signal for Δ160p53α, but also suggests that there was an evident difference between Δ160p53β and Δ160p53γ expression levels, a difference that is unbiased because both Δ160p53β and Δ160p53γ isoforms share a unique epitope for SAPU within the DNA-binding domain. Therefore, we hypothesized that the different expression of the Δ160p53γ isoform was caused by some issues linked to the pooled population of transfected H1299 cells. In order to analyze cell subgroups, we isolated four different populations expanded from five different Δ160p53γ-over-expressing H1299 single-cell clones. As shown in Figure S4C, by detecting with SAPU antibody the protein extracts of the pooled populations and of the single-cell Δ160p53γ clones, there were no significant differences in Δ160p53γ expression levels among the pooled Δ160p53γ-over-expressing H1299 cells and their single-clone sub-populations. Given these results, we decided to keep the pooled populations for all three Δ160p53-over-expressing H1299 cells for the following experiments.

**Figure 4:**
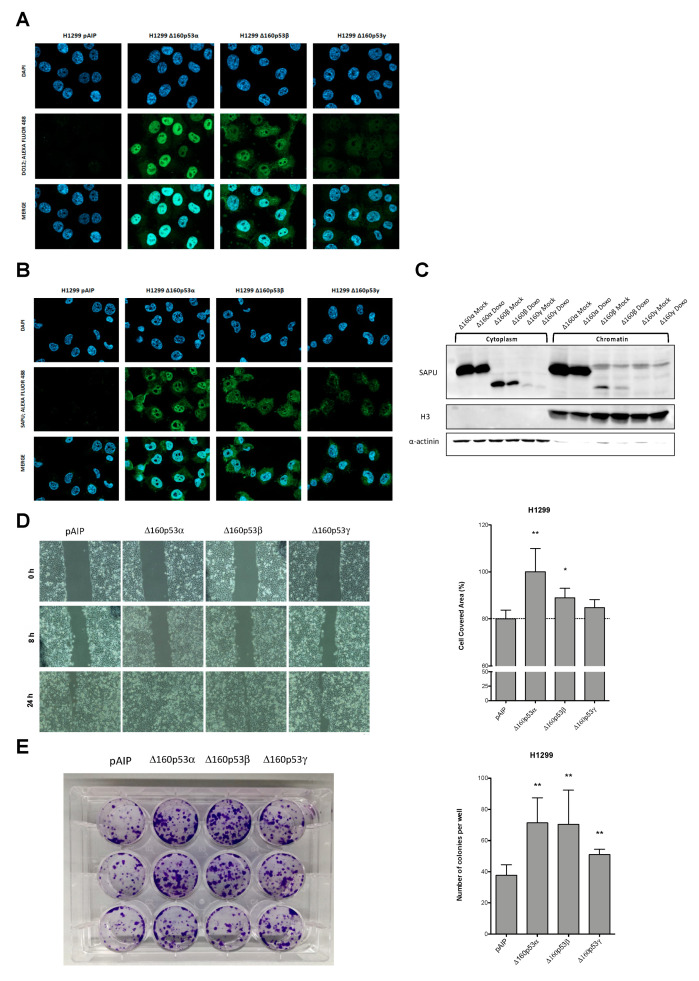
Localization and pro-oncogenic functions of Δ160p53 isoforms. (**A**,**B**) Immunofluorescence analysis of the subcellular localization of Δ160p53 proteins in H1299 cells stably over-expressing Δ160p53α, Δ160p53β, and Δ160p53γ isoforms, with the use of the p53 pantropic antibodies DO-12 (panel **A**) and SAPU (panel **B**). DAPI was used to counterstain the nuclei. (**C**) Cellular fractionation followed by Western blot of H1299 cells stably over-expressing Δ160p53 with or without 1.5 µM doxorubicin treatment for 24 h. SAPU antibody was used to detect the Δ160p53 isoforms. α-actinin was used as a loading control for the cytoplasmic protein fractions, whereas Histone H3 was used for the chromatin-bound protein fractions. (**D**) To measure the migration potential of H1299 stably over-expressing Δ160p53α, Δ160p53β, and Δ160p53γ, compared with the parental H1299 cells, we performed a wound-healing assay. Images were taken at different time points: 0, 8, and 24 h post scratch. Quantification of the migration potential after 24 h was obtained with the Wimasis Image Analysis tool. (**E**) The proliferation ability of H1299 clones stably over-expressing Δ160p53 isoforms compared with the empty control was evaluated with the colony formation assay. A total of 1000 cells was seeded into 6-well plates and left growing for 2 weeks. Visible clones were stained with crystal violet and manually counted. Bars in panels D and E represent the averages and the standard deviations of 3 biological experiments. ** = *p* < 0.01; * = *p* < 0.05.

After the establishment of H1299 cell lines stably over-expressing Δ160p53 variants, we wanted to examine, in depth, the possible analogies or differences in cellular localization. In order to do so, we performed immunofluorescence analysis, comparing two pantropic antibodies, DO-12 (used in the experiment reported by Marcel and colleagues [46]) and SAPU, and we further analyzed the distribution of Δ160p53 isoform within the cell through cellular protein fractionation and Western blot analysis. In Figure 4A, DO-12 antibody was used to detect p53 isoforms. The resolution used did not allow us to appreciate the previously observed perinucleolar pattern of Δ160p53α or the presence of foci in Δ160p53β-over-expressing cells [46], but we could clearly see a stronger nuclear localization for the Δ160p53α isoform and a less nuclear, more cytoplasmic accumulation of both Δ160p53β and Δ160p53γ. Of note, validating our previous observation about the Δ160p53γ variant expression levels, in order to detect this isoform, we needed to increase the exposure time up to 5500 ms, starting from 1500 ms used for Δ160p53α and Δ160p53β acquisitions. In panel B, with SAPU, we were able to observe a nuclear localization regarding Δ160p53α and an equally distributed nuclear/cytoplasmic localization of both Δ160p53β and Δ160p53γ. The presence of foci within the cells can be pointed out both in Δ160p53β- and γ-isoform-over-expressing cells: in the first case, the foci appeared to locate inside the nuclei; in the second case, they were strongly cytoplasmic, but perinuclear organized. Noteworthy is that SAPU antibody performed better in detecting the whole group of Δ160p53 variants (Figure 4B); differently to what we saw in panel 4A, using SAPU we could appreciate the signals coming from all three Δ160p53 isoforms by keeping the same exposure time of 1000 ms. As before, lower expression levels of Δ160p53γ were observed. Additionally, we examined Δ160p53 isoform localization within the cell by isolating proteins from the cytoplasm, from the nucleoplasm, or bound to the chromatin. While all three variants accumulated inside the cytoplasm, only Δ160p53α and, even if in small traces, Δ160p53β isoforms seemed to actually be imported inside the nucleus and bind to the chromatin (Figure S4D). This could be due to the presence of a proper oligomerization domain only in the α domain that allows the formation of p53 complexes, thus stabilizing the protein–DNA interactions. It was recently hypothesized that the presence of other peculiar domains also in β and γ variants could possibly induce the formation of stable protein complexes, but their position and the mechanism of action is still poorly known. Moreover, considering the ability of FL p53 to enter the nucleus in response to DNA damage, we wanted to evaluate possible differences in the ability of Δ160p53 isoforms to bind to the chromatin, comparing doxorubicin-treated cells and untreated cells. As shown in Figure 4C, we observed that the treatment with doxorubicin reduces the amount of all Δ160p53 isoforms, an effect that could be more visible with the chromatin recruitment of Δ160p53β.

To better understand the functions of Δ160p53 isoforms, we performed migration (wound healing) and proliferation (colony formation) assays in H1299 cells stably over-expressing these isoforms. Interestingly, the results demonstrate that Δ160p53α and β isoforms increased H1299 cell migration in vitro (Figure 4D). Moreover, the over-expression of each Δ160p53 isoform stimulated H1299 cell proliferation, even if the effect was less evident for Δ160p53γ (Figure 4E).

### 3.4. While BRAF Inhibitor Vemurafenib Has Little Effect on p53 Family Isoform Expression, Vemurafenib-Resistant Melanoma Cell Lines Show Altered Levels of Specific Isoforms

We tested the sensitivity of cellular models harboring the *BRAF V600E* mutation to vemurafenib (PLX-4032, abbreviated in some figures as PLX) to check its impact on the expression of p53 isoforms. Since BRAF signaling is mediated by the MAPK cascade, leading to the phosphorylation of ERK1/2 proteins, we used phospho-ERK as a sensor for vemurafenib efficacy. As visible in Figure S5A–E, we distinguished cell lines that were sensitive (Ma-Mel-61b, WM793B, Ma-Mel-55, Ma-Mel-86c, A375M, and WM278), partly resistant (Ma-Mel-61f), and resistant (Ma-Mel-54 and Ma-Mel-86a) to vemurafenib. Moreover, to further confirm the viability associated with the previously shown effect [32,33], we performed a cytotoxicity assay with MTT. The results confirm the previous observations, even if with a smaller effect, potentially depending on the different sensitivity of the used approaches (Figure S5F,G).

Once appropriate doses of vemurafenib were defined, we treated the cell lines to evaluate any changes in the endogenous expression of p53 isoforms. Unfortunately, in most cases the treatment with vemurafenib did not affect the expression of p53 isoforms (Figure S6). However, an appreciable reduction in Δ160p53α levels was visible in Ma-Mel-54, -55, and 86a (Figure S6A,B,D,E).

**Figure 5:**
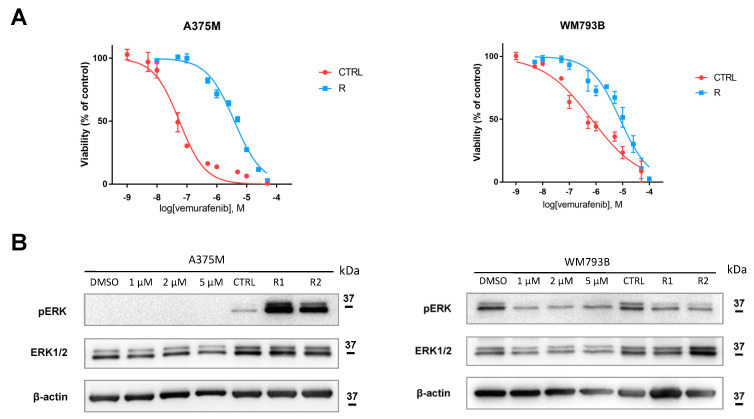
The sensitivity of parental and resistant cell lines to vemurafenib. (**A**) Viability of control parental cells (CTRL) and the corresponding sublines of vemurafenib-resistant A375M and WM793B cells (R) treated with increasing concentrations (0.01–100 μM) of vemurafenib for 72 h, determined by MTT assay. (**B**) ERK phosphorylation in parental cells after 24 h treatment with vemurafenib (1 µM, 2 µM, or 5 µM) or DMSO, as well as in untreated parental, control (CTRL), or resistant (R) lines, was evaluated using Western blot analysis. β-actin was used as a loading control. Representative results of at least three experiments (three biological replicates) are shown.

Based on these results, we hypothesized that an altered expression of p53 isoforms might be involved also in the resistance to BRAF inhibitors. Therefore, we developed two vemurafenib-resistant melanoma cell lines, A375M-R and WM793B-R (both *BRAF* V600E/*TP53* wt). After two months of treatment as described above, IC50 was shown to be 97- and 159-fold higher compared to the parental WM793B and A375M cell lines, respectively (Figure 5A). Likewise, to confirm that the cells had acquired resistance to vemurafenib, we determined the ERK phosphorylation (Figure 5B). A375M-R cells display a higher level of resistance in terms of both higher IC50 values and prevalence of phospho-ERK expression (Figure 5). In WM793B-R cell subline we noticed increased phosphorylation of ERK compared to parental cells treated with vemurafenib. Apparently, the treatment with vemurafenib inhibits BRAF signal transduction to the ERK protein in both cell lines tested, and reactivates this signaling pathway after acquiring resistance to vemurafenib in the A375M cell line only (Figure 5B).

**Figure 6:**
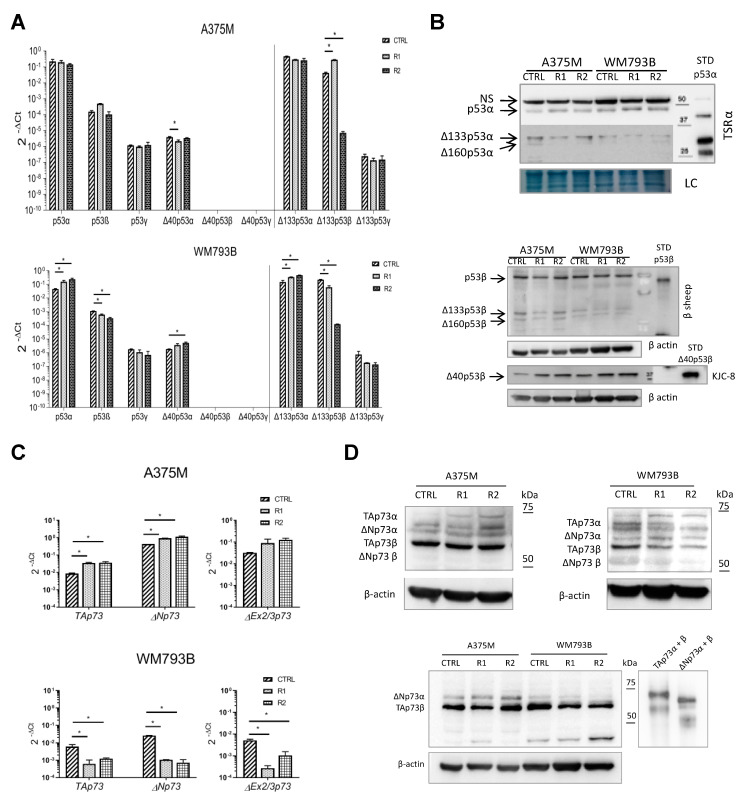
The expression of p53/p73 isoforms in parental and vemurafenib-resistant melanoma cell lines A375M-R and WM793B-R. (**A**) Expression of the *TP53* gene in the vemurafenib-resistant melanoma cell lines. Relative expression levels of p53 isoforms analyzed by pre-amplification followed by qPCR. The expression was normalized to the expression level of total *TP53*. (**B**) Western blot analysis of the endogenous p53 expression in parental and vemurafenib-resistant melanoma cell lines. p53 isoform expression was revealed using isoform-specific antibodies: TSRα and 421 to detect p53α isoforms; β-sheep and KJC8 to detect p53β isoforms. Naphthol blue staining or β-actin were used as loading controls. (**C**) Relative expression levels of *TP73* isoforms were analyzed by qPCR. The expression was normalized to the geometric mean of GUSB and TBP. (**D**) Western blot analysis of p73 isoform expression in vemurafenib-resistant melanoma cell lines was revealed using pantropic rabbit polyclonal EP436Y anti-p73 antibody. β-actin was used as a loading control. (**A**,**C**) Average of two independently performed experiments ± SD is shown. One-way Anova with a post hoc Tukey–Kramer test was used. * = *p*-value < 0.05. (**B**,**D**) Representative data of three independent experiments yielding similar results are shown.

The change in the gene and protein expression profile of p53/p73 isoforms was evaluated by qPCR and Western blot analysis in both vemurafenib-resistant cell lines (Figure 6A–D). Although statistical analysis of *TP53* gene expression showed a significant difference in the gene expression of most isoforms between parental (CTRL) and resistant (R) lines, all three biological replicas had in common only the different expression of the Δ133p53β isoform, which was reduced in subline R2 compared to R1 and control parental cells, in both A375M and WM793B (Figure 6A). Expression at the protein level, as well as at the gene level, was checked in three biological replicates. Using α- and β-specific anti-p53 antibodies, we noticed a slight decrease in Δ133p53α and β expression in A375M-R1 and in vemurafenib-resistant WM793B cells. The expression of Δ40p53β was elevated in both A375M and WM793B resistant cell lines (Figure 6B; Figure S6H).

The expression of the *TP73* gene is shown in Figure 6C. Interestingly, in vemurafenib-resistant WM793B sublines, the expression of all tested isoforms of the *TP73* gene (TAp73, ΔNp73, and ΔEx2/3p73) was reduced, while in vemurafenib-resistant A375M sublines, their expression was increased. The protein expression (Figure 6D; Figure S6H) confirmed these results. We detected a decrease in the expression of the TAp73β isoform in WM793B-R cells, as well as of ΔNp73α and β compared to the control cells. In contrast, in metastatic A375M-R cells an increase in the expression of the TAp73α and ΔNp73α isoforms was observed.

## 4. Discussion

Melanoma is an extremely aggressive type of skin tumor that arises from melanocytes. Although relatively rare, it is responsible for the vast majority of skin tumor-related deaths. We previously reported p53 and p73 gene and protein expression profiles in a subset of melanoma patient samples [30]. Here, we analyzed p53 and p73 expression in a panel of melanoma cell lines in basal conditions, and in response to common anti-cancer therapies, including standard DNA-damaging agents such as doxorubicin, etoposide, cisplatin, and radiotherapy, as well as drugs used prevalently in melanoma, such as dacarbazine and the melanoma-specific drug vemurafenib. It was previously reported that low-molecular-weight p53 isoforms were expressed in several malignancies [35,47,48,49,50,51,52], including melanoma [29,30]. To assess the gene expression of *TP53,* we employed a sophisticated approach in which qPCR was performed as a nested reaction following initial RT-PCR amplification, and detected a high expression of p53α and ∆133p53α isoforms, followed by p53β, ∆40p53α, and ∆133p53β, respectively. Notably, there was no association between gene expression with p53 mutation status, presence of BRAF V600E mutation, or metastatic potential. In Western blot analysis using different antibodies, we were able to determine the expression of six p53 isoforms: p53α, p53β, ∆40p53α, ∆133p53α, ∆133p53β, and ∆160p53α, whose expression was diverse compared with primary melanocytes. As expected, mut p53 cells showed generally higher protein expression of p53 isoforms, particularly mut p53α, which is more stable than wt. The BRAF mutation status does not affect the expression of the p53 isoforms at either the gene or protein level.

In our previous clinical study [30], we reported a relatively high expression of all isoforms, except Δ40p53β, which was significantly downregulated in tumor tissue. However, this was not confirmed at the protein level, where ∆133p53α and ∆160p53α showed statistically significantly higher expression in tumors. Another study on melanoma cell lines described that p53β and Δ40p53 are more expressed than p53α, compared to fibroblasts and melanocytes, suggesting that their expression may play a role in melanoma development [29].

A homologue of *TP53*, the *TP73* gene, is deregulated in cancer, primarily through upregulation of ΔNp73 isoforms. We were able to determine the gene expression of three groups of cancer-relevant *TP73* isoforms, TAp73, ∆Np73, and the ΔEx2/3p73 isoform, the latter of which was found to be upregulated in metastatic melanoma and correlated with multidrug-resistance genes [25,26]. We found a higher expression of N-terminally truncated isoform ∆Np73 than TAp73. Additionally, the expression of TAp73 was the highest in the BRAF wt/p53 wt cell line Mel501. There is a lack of studies analyzing p73 isoform gene expression in melanoma. Tuve and colleagues detected the over-expression of both TAp73 and ∆Np73 variants ΔEx2p73 and ΔEx2/3p73 (spliced transcripts derived from the first promoter) as an important event of melanoma progression, which activates the epithelial-to-mesenchymal transition (EMT), as well as pluripotency and stemness-like characteristics [25,53,54]. In addition, when melanoma cells over-expressing ΔNp73 were injected into mice, they developed depigmented tumors due to loss of active tyrosinase, which reactivates the EMT signaling cascade, a mesenchymal-like cell phenotype, and increased invasiveness [55]. On the other hand, ΔNp73, derived from promoter P2, was the predominant isoform in benign nevi [25]. This observation is consistent with our recent research, in which we unexpectedly found a higher expression of FL TAp73 isoforms than ΔNp73 in metastatic melanoma. Moreover, ΔNp73 expression was significantly downregulated in metastatic melanoma clinical samples compared to healthy tissue [30]. The research conducted by Tuve and collaborators was confirmed by Sakil and colleagues, who detected ΔEx2/3p73 expression in melanoma patient samples using qRT-PCR, but not P2-derived ΔNp73 [26]. Notably, at the protein level we were able to detect four p73 isoforms, TAp73α, TAp73β, ΔNp73α, and ΔNp73β. The most prominent was the expression of TAp73β, followed by ΔNp73α in almost all cell lines regardless of the *TP53* or *BRAF* mutational status. Unfortunately, there is a paucity of studies exploring the p73 isoform protein expression due to the lack of isoform-specific antibodies. So far, protein expression has been analyzed only using immunohistochemistry, and an over-expression of total p73 has been found in invasive cutaneous melanoma [56]. The observed inconsistencies between gene and protein expression are expected, since post-transcriptional regulation plays an important role in the modification of protein levels.

Very interestingly, the ∆160p53α isoform was the most variable in expression and was subject to modification by DNA-damaging agents in melanoma cell lines. To date, only two reports have focused on the ∆160p53 isoform [46,57], neither of which in melanoma. For these reasons, we analyzed more deeply the localization and functions of ∆160p53 isoforms by generating clones of H1299 p53 null cells over-expressing ∆160p53 isoforms. Consistent with Marcel and colleagues [46], we confirmed the nuclear localization of ∆160p53α and the presence of ∆160p53β nuclear and cytoplasmic foci, this time with stable over-expression clones (Figure 4A,B). Notably, with the SAPU antibody, we were able to detect the discrete perinuclear foci of the ∆160p53α isoform (Figure 4B), which has never been reported so far. In addition, we were also able to demonstrate for the first time the localization of the ∆160p53γ isoform within cytoplasmic foci (Figure 4B). Moreover, we confirmed the ability of ∆160p53α, and to a lesser extent ∆160p53β, to be recruited on chromatin (Figure 4C). Further studies are needed to understand the role of ∆160p53 isoforms in the regulation of transcription and the relevance of the observed enrichment in nuclear and cytoplasmic foci. Lastly, we observed a potential role of high levels of ∆160p53 isoforms in cancer aggressiveness, particularly proliferation and migration (Figure 4D,E). However, we could not exclude the possibility that cell proliferation might participate in the observed increased migration. These results are relevant, since a higher expression level of ∆160p53 isoforms could play a significant role in cancer and influence p53-dependent responses. A function in invasion was reported in a unique study for ∆160p53α [57], but this is the first time these isoforms have been linked to increased survival and proliferation. Nonetheless, we have to mention that these last results were obtained in H1299 cells, which, despite being the purest cellular model to study the individual contribution of single p53 isoforms, are derived from lung cancer. Therefore, these effects need further investigation in the future.

A significant advance in treating melanomas harboring a mutant version of BRAF was the discovery of targeted therapy based on MAPK signaling pathway inhibitors. The milestone was the approval of the mut BRAF protein inhibitor (BRAFi), vemurafenib (Zelboraf, PLX4032), in 2011 [58]. Shortly thereafter, the inhibitors dabrafenib [59] and encoraphenib [60] were also approved. Although BRAFi initially showed excellent results in tumor reduction and withdrawal, long-term success was not possible due to the rapid appearance of tumor resistance to therapy. In parallel with the development of BRAFi, MEK protein inhibitors (MEKi) have also been developed. The first approved MEKi was trametinib in 2013, followed by cobimetinib and binimetinib. Although BRAFi/MEKi combination therapy has advantages over BRAFi monotherapy, resistance still represents a significant problem [61]. Acquired resistance involves mainly the reactivation of the MAPK pathway, the stimulation of phosphatidylinositol-3-kinase (PI3K) activity, and the persistent firing of tyrosine kinase receptors [62,63,64]. Based on this, we evaluated whether exposure to vemurafenib might alter the expression pattern of p53 isoforms. As expected, given this molecule does not have an impact on DNA damage or other processes known to stabilize p53, we did not observe any changes in the endogenous expression of p53 isoforms in melanoma cell lines after treatment, except the reduction in Δ160p53α levels in Ma-Mel-54, -55, and -86a (Figure S6).

However, because of their distinct roles in carcinogenesis, p53/p73 isoforms might be involved in developing resistance to targeted therapy in metastatic melanoma due to their aberrant expression, localization, and/or mutual inhibitory protein interactions. For this reason, we developed and characterized two melanoma cell lines, WM793B and A375M, resistant to vemurafenib. After checking viability upon exposure to vemurafenib, we determined the ERK phosphorylation to confirm that the cells had acquired resistance. The vemurafenib-resistant metastatic melanoma cell line A375M-R displays higher IC50 values and a prevalence of phospho-ERK expression compared to parental cells (Figure 5). Although in primary melanoma cells resistant to vemurafenib, WM793B-R, we noticed increased phosphorylation of ERK compared to parental cells treated with vemurafenib, there was a prevalence of unphosphorylated ERK. Therefore, we assume that vemurafenib treatment inhibits the mutated BRAF protein, thereby stopping signal transduction to the ERK protein, but does not reactivate the MAPK signaling pathway after acquiring vemurafenib resistance. This implies that another mechanism contributes to vemurafenib resistance in the WM793B cell line, possibly the activation of the PI3K/AKT signaling pathway. Apparently, in metastatic melanoma cell line A375M, treatment with vemurafenib inhibits BRAF, thereby stopping signal transduction to the ERK protein, and it reactivates this signaling pathway after acquiring resistance to vemurafenib (Figure 5B).

Since MAPK (Ras-Raf-MEK-ERK) and p53 signaling pathways are closely related and jointly participate in regulating tumor treatment resistance, as described in many scientific publications [65,66,67], we explored the involvement of the p53 family proteins in the emergence of vemurafenib resistance. Therefore, we analyzed their expression at the protein and gene levels. By analyzing the expression of the *TP53* gene, we found a significant decrease in the Δ133p53β isoform with the appearance of vemurafenib resistance in the R2 sublines (grown in the presence of vemurafenib for 10 months) of WM793B and A375M cells (Figure 6A). The Δ133p53β isoform has been associated with tumor invasion, increased cancer stem cell potential, and generally a poor outcome [68,69]. This phenotype was confirmed by our previous study, where we correlated the high expression of p53α and Δ133p53β isoforms at the mRNA level in clinical melanoma samples with reduced patient survival [30]. However, in our melanoma cells the vemurafenib resistance led to a decrease in the expression of the Δ133p53β isoform at the mRNA level. The protein analysis of p53 isoform expression did not fully follow the results obtained by gene expression analysis (Figure 6B; Figure S6H). Nevertheless, a slight decrease in Δ133p53β protein expression was detected in vemurafenib-resistant cells. Furthermore, the expression of Δ40p53β was increased in both A375M and WM793B resistant cell lines. It was demonstrated that the role of Δ40p53 depends on the cellular context [70]. In melanoma cells, Δ40p53 represses p21WAF1 and PUMA expression when it is more expressed than p53, both at the basal level as well as in response to DNA-damaging agents. However, Δ40p53 had no transcriptional activity in the absence of p53 on these genes [29]. Therefore, the Δ40p53:p53 ratio is an essential factor in controlling the p53 response [71].

In vemurafenib-resistant primary melanoma cells, WM793B-R, the gene expression of both TAp73 and ΔNp73 isoforms was decreased, while in vemurafenib-resistant metastatic cells, A375M, it was increased (Figure 6C). The protein expression results obtained by Western blot analysis mainly match the gene expression (Figure 6D; Figure S6H). Interestingly, in WM793B-R cells we observed a reduction in the expression level of the TAp73β isoform, which is the most transactivating among the p73 isoforms, with tumor-suppressive functions [38,72,73]. This event could be considered as a pro-survival and pro-aggressiveness advantage for resistance cells. The obtained results confirm our assumption that p53 family members are involved in the acquired resistance to vemurafenib. However, further research should determine whether they are the cause or the consequence of this phenomenon.

In summary, with this study, we can propose the expression level of Δ160p53 as informative in cancer patients (and specifically in melanoma), given that its higher levels can be associated with features of cancer aggressiveness. Moreover, in general, we can suggest that the analysis of p53 family members might be considered clinically relevant in a personalized medicine view of patient management, as changes in the expression levels of specific isoforms (i.e., high Δ40p53β or low TAp73β) can be informative regarding therapy effectiveness. Lastly, by knowing which oncogenic p53 isoform is highly expressed in cancer tissues, we can hypothesize the potential use of small molecules to block alternative translation initiation or reduce the specific mRNA amount.

## Data Availability

Data are contained within the article and Supplementary Materials.

## Supplementary Materials

The following Supplementary Materials are available online at www.mdpi.com/article/10.3390/cancers13205231/s1. Table S1. List and characteristics of melanoma-derived cell lines, Figure S1. Position of primers used to quantify the gene expression of *TP53* isoforms, Figure S2. FASAY assays on melanoma cell lines with uncertain *TP53* status, Figure S3. p53 isoforms are differentially expressed in melanoma cell lines, Figure S4. Generation of H1299 cell lines stably over-expressing Δ160p53α, Δ160p53β and Δ133p53γ, Figure S5. Evaluation of the sensitivity to vemurafenib in several melanoma cell lines, Figure S6. Analysis of the effect of different anti-cancer agents on p53 isoforms’ expression, Figure S7. Original uncropped blots.
