# Supplementary material for: Altered Expression of Shorter p53 Family Isoforms Can Impact Melanoma Aggressiveness
Source: Cancers (Basel). 2021 Oct 18;13(20):5231. doi: 10.3390/cancers13205231 (PMC8533715; doi:10.3390/cancers13205231)
Supplement: Supplementary file 1 [file cancers-13-05231-s001.zip › cancers-1417131 Supplementary layout final.pdf]

cancers

MDPI

Supplementary Material: Altered Expression of Shorter p53 Family Isoforms Can Impact Melanoma Aggressiveness

Ana Tadijan, Francesca Precazzini, Nikolina Hanžić, Martina Radić, Nicolò Gavioli, Ignacija Vlašić, Petar Ozretić, Lia Pinto, Lidija Škreblin, Giulia Barban, Neda Slade and Yari Ciribilli

Table S1. List of the primary and metastatic melanoma cell lines with mutational status of p53 and BRAF.

	CELL LINES	Abbreviation*	MELAMOMA STAGE	STATUS p53	ClinVar p53	STATUS BRAF
PRIMARY	WM793B	CRL-2806	primary	wt	-	p.V600E
	WM278	CVCL_6473	primary	wt	-	p.V600E
	LM6 (4405P)	CVCL_WM23	primary	Null	-	wt
	Mel-505	CVCL_3976	primary	p.R273H	12366 P	wt
				p.P309S	458575 UC	
	Mel-224	CVCL_U915	primary	p.H179Y	127815 P/LiP	wt
	G-361	CVCL_122	primary	wt	-	p. V600E
METASTATIC	A375	CRL-7904	metastatic	wt	-	p.V600E
	A375M	CVCL_B222	metastatic	wt	-	p.V600E
	Mel-501	CVCL_4633	metastatic	wt	-	wt
	CHL-1	CRL-9446	metastatic	p.H193R	184979 LiP	wt
	SK-MEL-2	CVCL_0069	metastatic	p.G245S	12365 P	wt
	SK-MEL-3	CVCL_0550	metastatic	p.R267W	141764 LiP	p.V600E
	SK-MEL-5	CVCL_0527	metastatic	wt	-	p.V600E
	SK-MEL-24	CVCL_0599	metastatic	wt	-	p.V600E
	SK-MEL-28	CVCL_0526	metastatic	p.L145R	1027110 UC	p.V600E
	MeWo	CVCL_0445	metastatic	p.E258K	12348 P/LiP	wt
				p.Q317*	450344 P/LiP	
	RPMI-7951	CVCL_1666	metastatic	p.S166*	528239 UC	p.V600E
	WM266-4	CVCL_2765	metastatic	wt	-	p.V600D
	Ma-Mel-8a	Univ. Essen	metastatic	Het R283C	127824 UC	wt
	Ma-Mel-35	CVCL_A170	metastatic	wt	-	wt
	Ma-Mel-54a	CVCL_A189	metastatic	wt	-	p.V600E
	Ma-Mel-55	CVCL_A190	metastatic	wt	-	p.V600E
	Ma-Mel-61b	CVCL_C292	metastatic	wt	-	p.V600E
	Ma-Mel-61f	CVCL_S668	metastatic	wt	-	p.V600E
	Ma-Mel-86a	CVCL_A221	metastatic	p.R248W	12347 P	p.V600E
	Ma-Mel-86c	Univ. Essen	metastatic	p.R248W	12347 P	p.V600E

- Abbreviations from ATCC or Cellosaurus database
- P, Pathogenic; LiP, Likely pathogenic; UC, Uncertain
- p53 data from http://p53.iarc.fr/CellLines.aspx

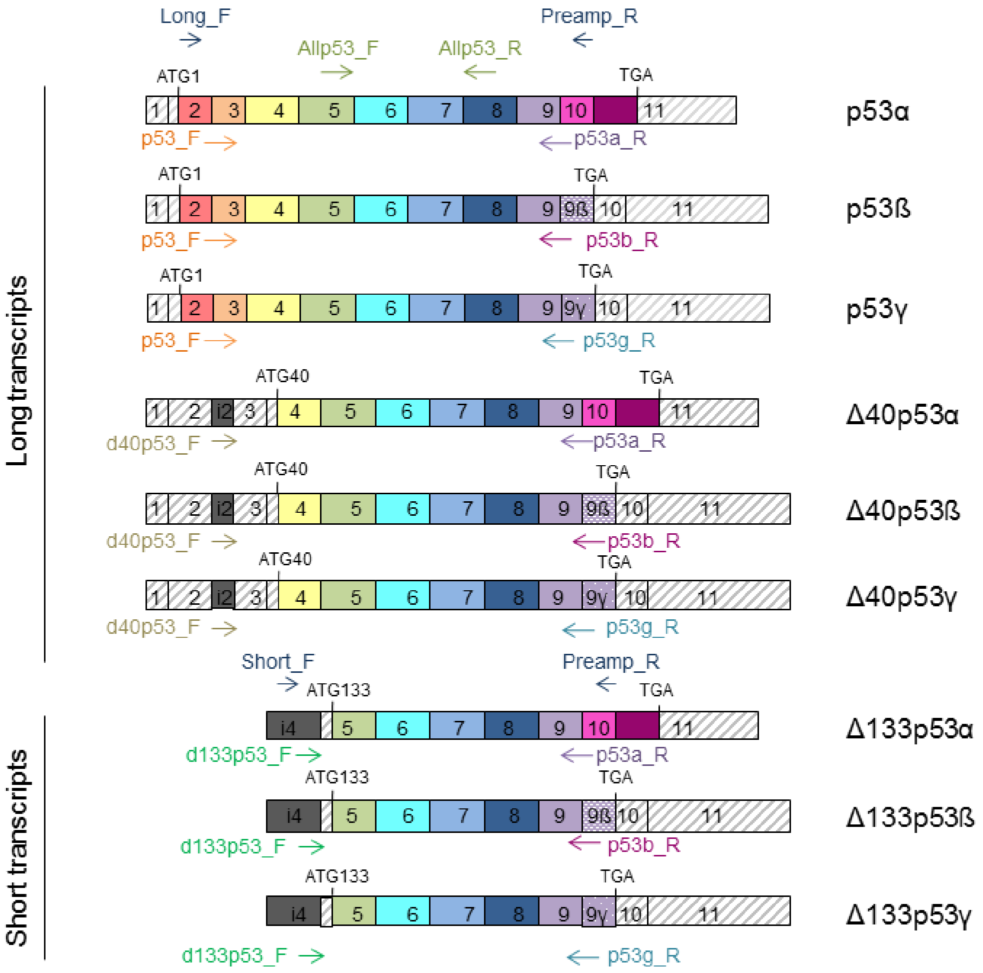

Figure S1. Position of primers used to quantify the gene expression of *TP53* isoforms. First, two separate PCR reactions were performed to distinguish "long" full-length and Δ40 from "short" Δ133 isoforms. Generated pre-amplified cDNA templates were used for nested qPCR with combinations of three forward and three reverse isoform-specific primers.

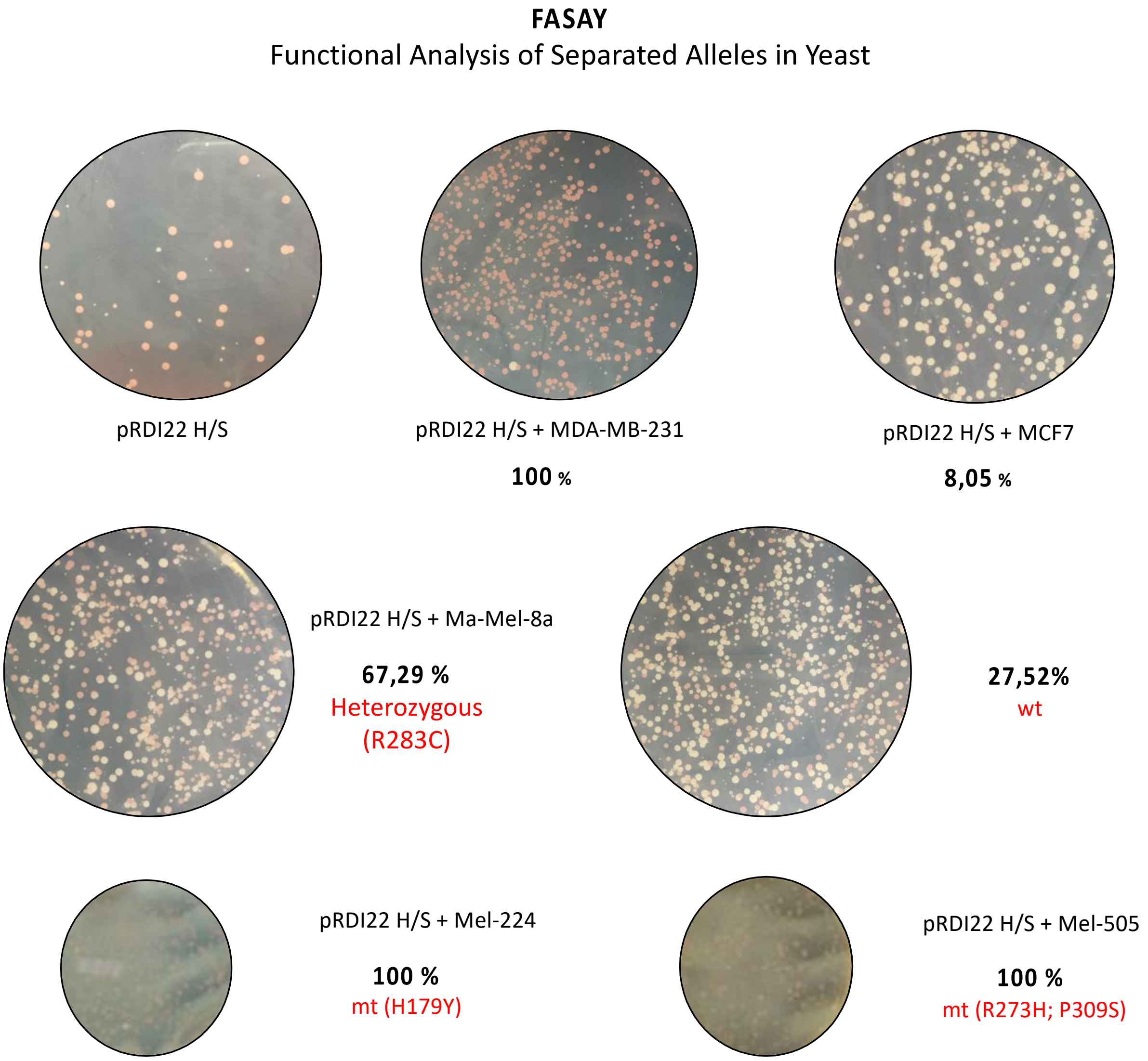

Figure S2. FASAY assays on melanoma cell lines with uncertain *TP53* status. FASAY (Functional Analysis of Separate Alleles in Yeast) assay was done for all the cell lines with a doubtful/unconfirmed *TP53* status. PCR on the cDNA from melanoma-derived cell lines was performed on *TP53* coding region with p53-P3 and p53-P4 primers. PCR products and linearized pRDI22 vector were co-transformed in yeast cells to allow for homologous recombination in vivo. The sample pRDI22 digested with Hind III and Stu I restriction endonucleases (pRDI22 H/S) was used as negative control and represented the background of red colonies. The analysis of MDA-MB-231 and MCF7 cells served as positive and negative controls for the presence of a mutant or wild-type p53, respectively. Indicated are the percentages of red colonies and the *TP53* status.

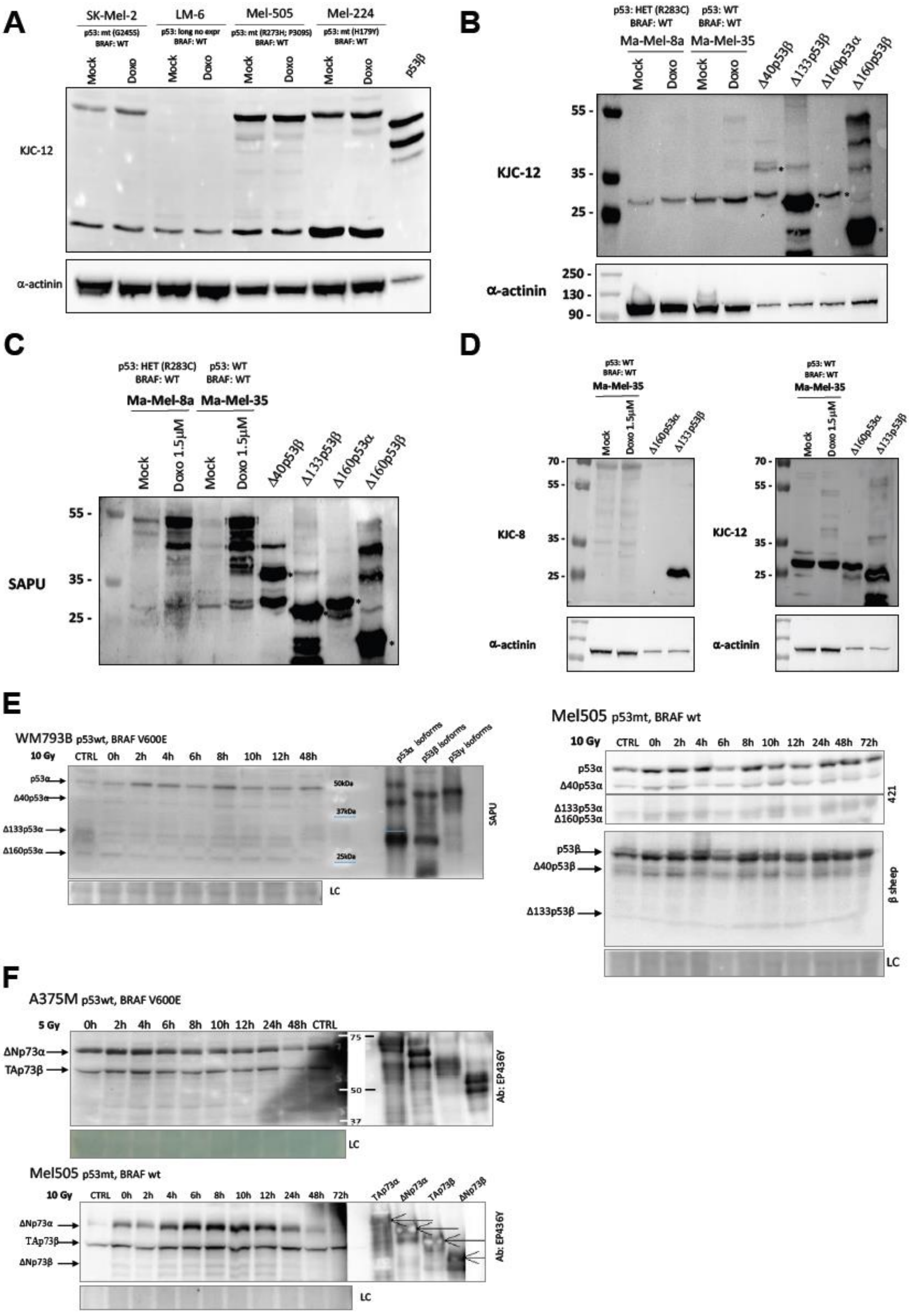
A
SK-Mel-2
p53: mt (G245S) BRAF: WT
LM-6
p53: long no expr BRAF: WT
Mel-505
p53: mt (R273H; P309S) BRAF: WT
Mel-224
p53: mt (H179Y) BRAF: WT
Mock Doxo Mock Doxo Mock Doxo Mock Doxo p53β
KJC-12
α-actinin
B
p53: HET (R283C) BRAF: WT
p53: WT BRAF: WT
Ma-Mel-8a
Ma-Mel-35
Mock Doxo Mock Doxo Δ40p53β Δ133p53β Δ160p53α Δ160p53β
KJC-12
55 - 35 - 25 -
α-actinin
250 - 130 - 90 -
C
p53: HET (R283C) BRAF: WT
p53: WT BRAF: WT
Ma-Mel-8a
Ma-Mel-35
Mock Doxo 1.5μM Mock Doxo 1.5μM Δ40p53β Δ133p53β Δ160p53α Δ160p53β
SAPU
55 - 35 - 25 -
D
p53: WT BRAF: WT
Ma-Mel-35
Mock Doxo 1.5μM Δ160p53α Δ133p53β
KJC-8
KJC-12
70 - 55 - 35 - 25 -
α-actinin
E
WM793B p53wt, BRAF V600E
10 Gy CTRL 0h 2h 4h 6h 8h 10h 12h 48h
p53α Δ40p53α Δ133p53α Δ160p53α
50kDa 37kDa 25kDa
p53α isoforms p53β isoforms p53γ isoforms
SAPU
LC
Mel505 p53mt, BRAF wt
10 Gy CTRL 0h 2h 4h 6h 8h 10h 12h 24h 48h 72h
p53α Δ40p53α Δ133p53α Δ160p53α
421
p53β Δ40p53β Δ133p53β
β sheep
LC
F
A375M p53wt, BRAF V600E
5 Gy 0h 2h 4h 6h 8h 10h 12h 24h 48h CTRL
ΔNp73α TAp73β
75 50 37
Ab: EP436Y
LC
Mel505 p53mt, BRAF wt
10 Gy CTRL 0h 2h 4h 6h 8h 10h 12h 24h 48h 72h
TAp73α ΔNp73α TAp73β ΔNp73β
ΔNp73α TAp73β ΔNp73β
Ab: EP436Y
LC

G

Figure 3C - upper panel A375M

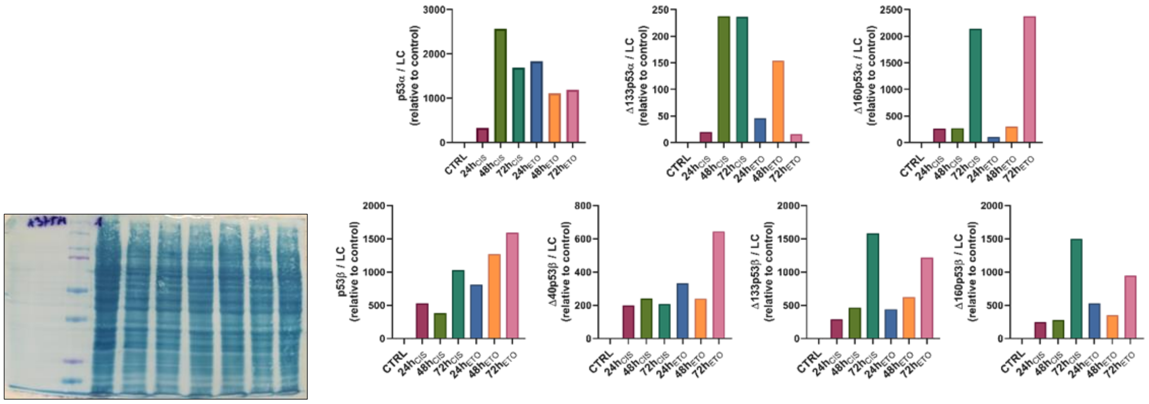

Figure 3C - lower panel WM793B

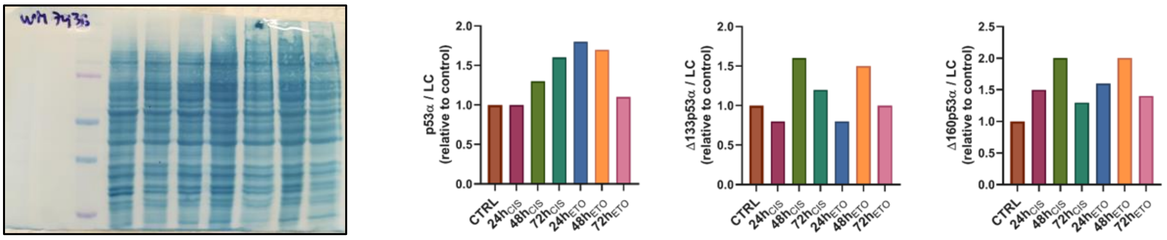

Figure 3D - upper panel A375M 5Gy

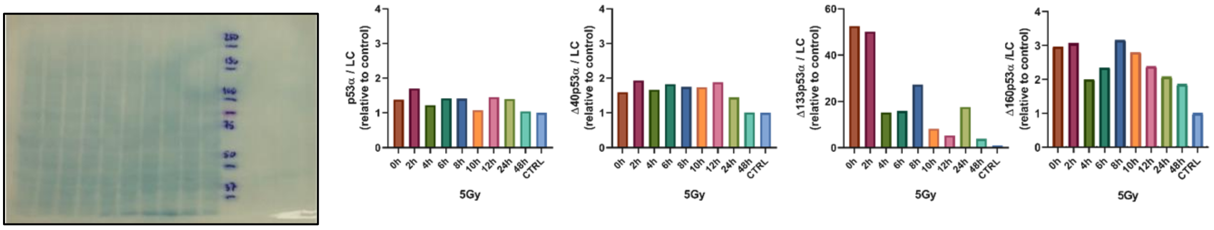

Figure 3D - lower panel A375M 10Gy

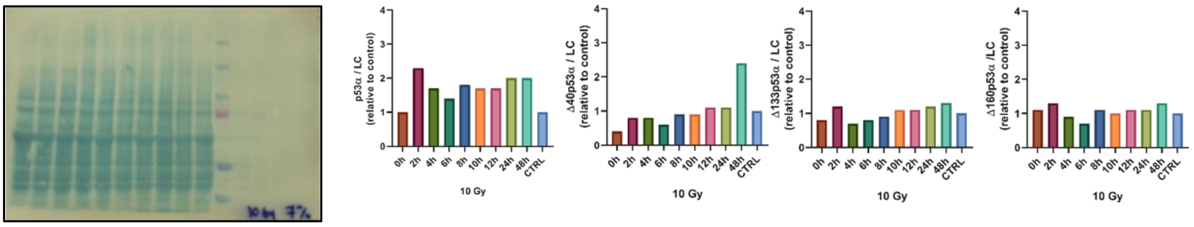

Figure S3. p53 isoforms are differentially expressed in melanoma cell lines in response to DNA damage. (**A**) An additional group of melanoma-derived cell lines was analyzed to determine the p53 isoforms expression levels upon treatment with 1.5 µM doxorubicin for 24 h. (**B-D**) Given the highly variability in expression of a low molecular weight p53 isoform among the different melanoma-derived cell lines, to pinpoint the nature of that p53 isoform, we performed western blot

using different pantropic (SAPU and KJC-12) as well as isoform-specific antibodies (KJC-8, β-specific) in additional cell lines Ma-Mel-8a and Ma-Mel-35 cells. To better visualize full length p53 isoforms cells were treated with 1.5 μM doxorubicin for 24 h. As a control we used transient over-expression of specific p53 isoforms in H1299 cells. (**E**) Two additional melanoma cell lines, WM793B, a p53 wt cell line, and Mel505, a p53 mut, were analyzed after γ-irradiation to determine p53 isoforms expression. To detect p53 isoforms in WM793B cells pantropic anti-p53 antibody (SAPU) was used. To distinguish α and β p53 isoforms after γ-irradiation in Mel505 cells, α- and β-specific anti-p53 antibodies (421 and β sheep, respectively) were used. (**F**) To detect the expression of p73 isoforms after γ-irradiation in two melanoma cell lines, we used pantropic anti-p73 antibody (EP436Y). (**G**) Naphthol blue stained membranes and quantification of the expression of p53/p73 proteins shown in Figure 3C-D using Image Lab software 6. The expression of p53/p73 proteins was normalized to the intensity of the naphthol blue membrane staining of the same samples. Three random strips of naphthol blue staining (a representative one is shown in the Figure 3C,D) were quantified and their sum was used for normalization of the expression of a protein of interest.

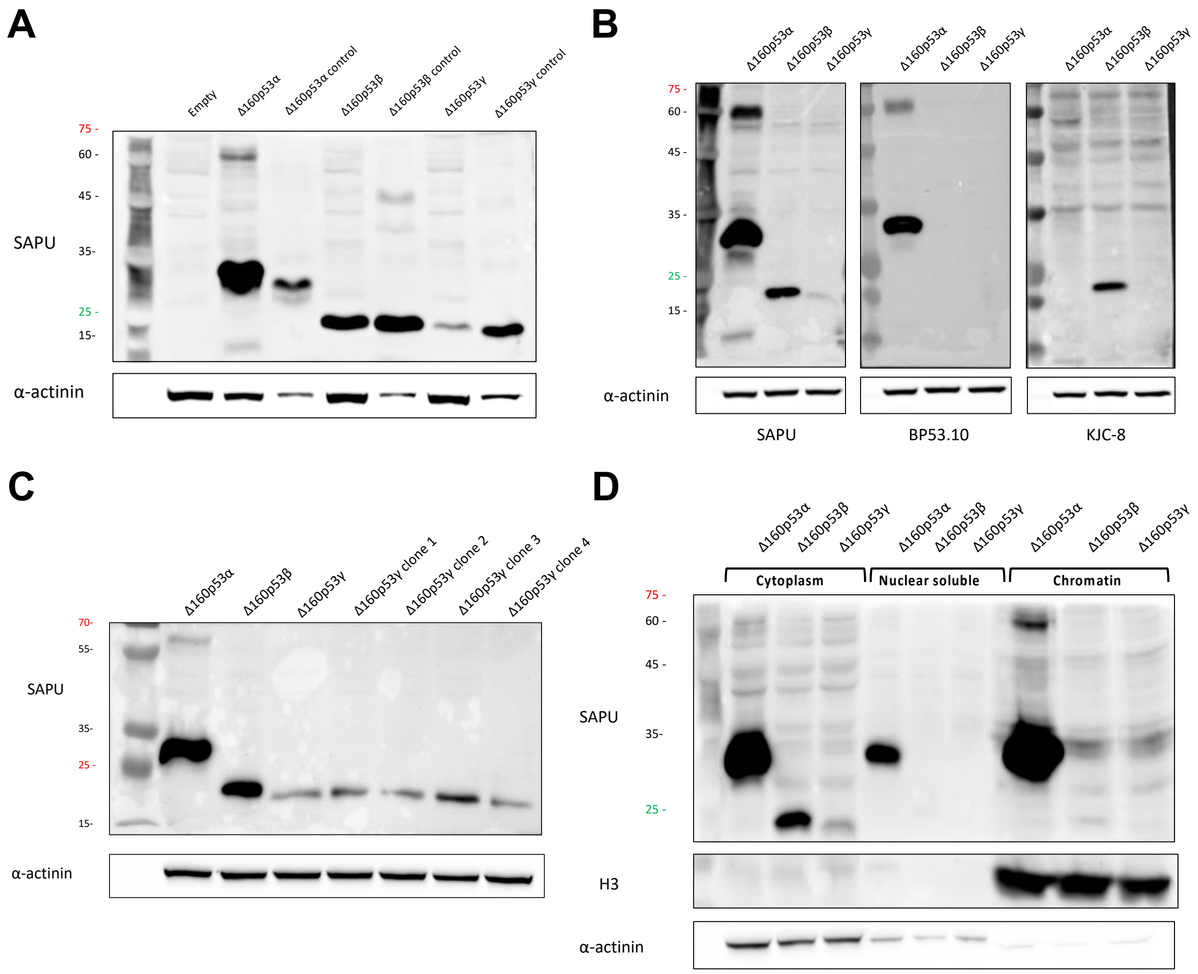

Figure S4. Generation of H1299 cell lines stably over-expressing Δ160p53α, Δ160p53β and Δ133p53γ. (**A**) Western blot analysis with SAPU antibody on protein extracts from H1299 cells stably transfected with the indicated Δ160p53 isoforms. As a control we used transient transfection in H1299 cells. (**B**) To confirm α or β specificity, protein extracts from panel A were also detected with α-specific (BP53.10) and β-specific (KJC-8) antibodies. (**C**) Western blot analysis with SAPU antibody on different single cell H1299 clones stably over-expressing Δ160p53γ isoform to check putative variability in expression among clones. α-actinin was used as loading control in all the blots. (**D**) Cellular fractionation of H1299 cells stably over-expressing Δ160p53 isoforms. SAPU antibody was used to detect the Δ160p53 isoforms; α-actinin was used as loading control for the cytoplasmic protein fraction whereas Histone H3 for the chromatin-enriched protein fraction.

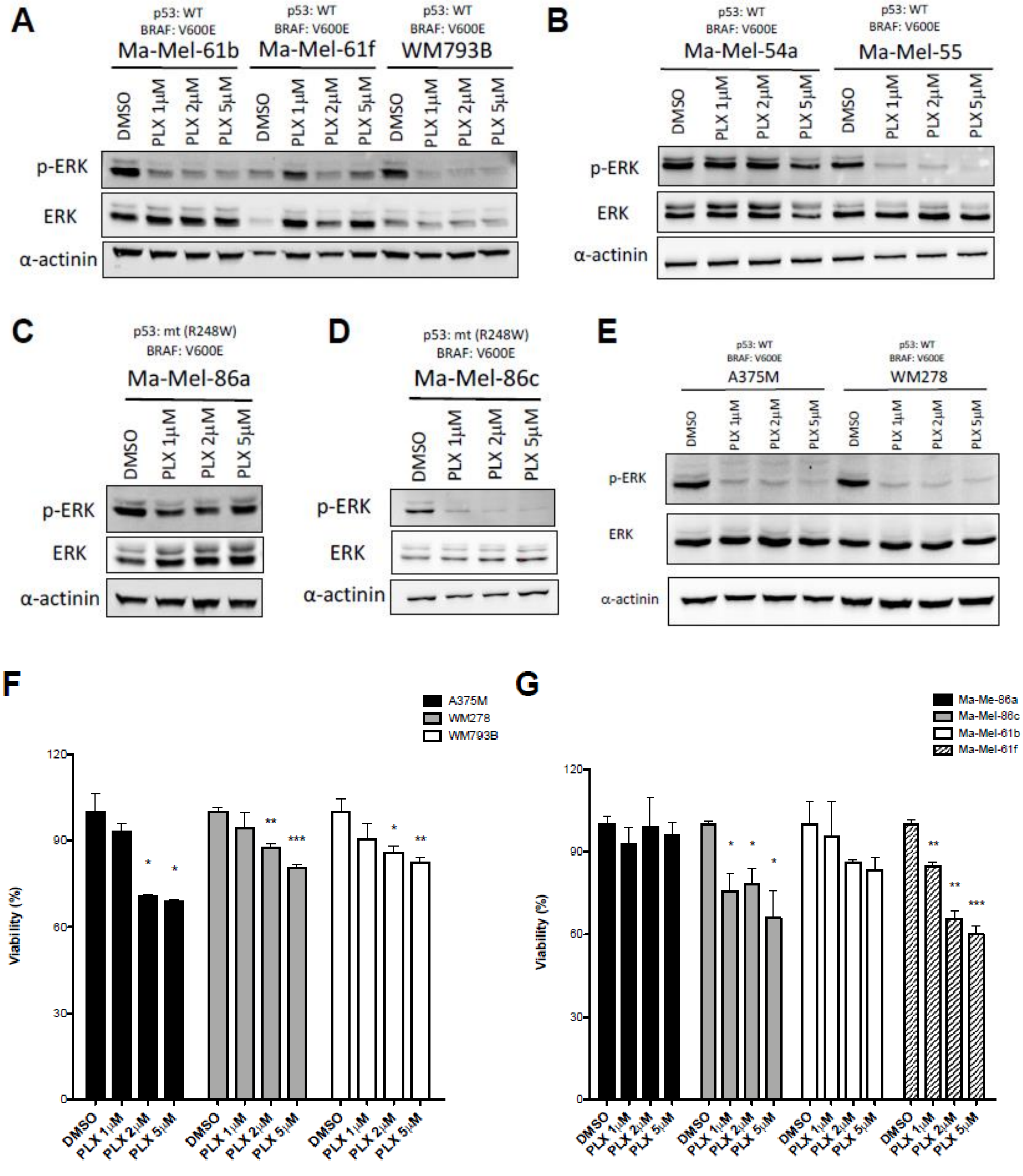

Figure S5. Evaluation of the sensitivity to vemurafenib in several melanoma cell lines. In order to evaluate the sensitivity of different melanoma cell lines to PLX-4032, cells were treated with increasing concentration of PLX-4032 (1, 2, and 5 µM) for 24 hours. (**A-E**) As a readout of the sensitivity to PLX-4032, we detected by Western blotting the phosphorylated form of ERK in comparison with total ERK protein. DMSO was used as a control and α-actinin as reference protein. (**F, G**) Alternatively, the viability of most of the cell lines analyzed with Western blotting was measured with MTT assay upon the treatment with PLX-4032 (1, 2, and 5 µM) for 48 hours. Presented are the averages and the standard deviations of 3 biological experiments. * = p < 0.05, ** = p < 0.01, *** = p < 0.001, t-student test.

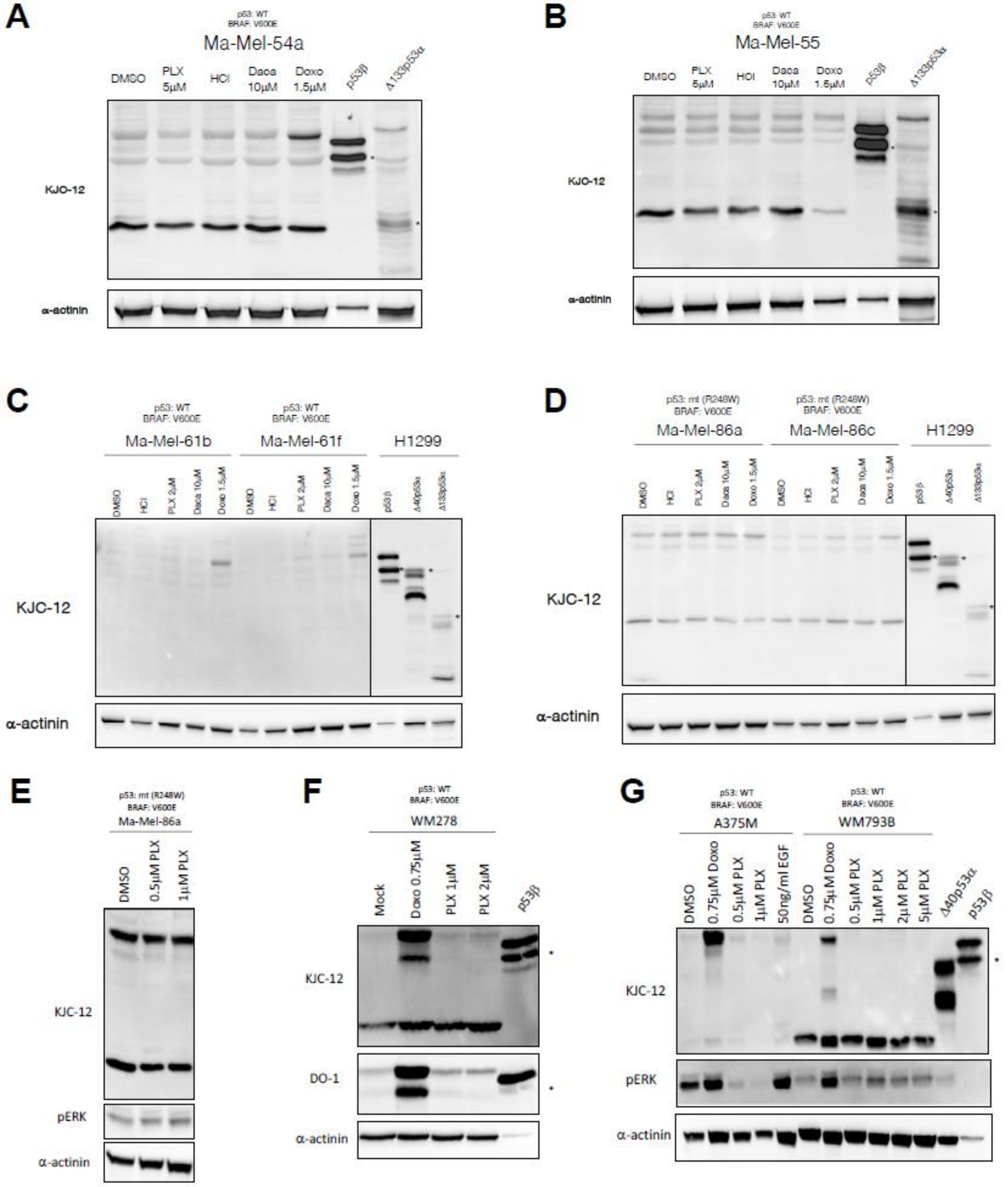
A
p53: WT
BRAF: V600E
Ma-Mel-54a
DMSO
PLX 5µM
HCl
Daca 10µM
Doxo 1.5µM
p53β
Δ133p53α
KJC-12
α-actinin
B
p53: WT
BRAF: V600E
Ma-Mel-55
DMSO
PLX 5µM
HCl
Daca 10µM
Doxo 1.5µM
p53β
Δ133p53α
KJC-12
α-actinin
C
p53: WT
BRAF: V600E
Ma-Mel-61b
p53: WT
BRAF: V600E
Ma-Mel-61f
H1299
DMSO
HCl
PLX 2µM
Daca 10µM
Doxo 1.5µM
p53β
Δ40p53α
Δ133p53α
KJC-12
α-actinin
D
p53: mt (R248W)
BRAF: V600E
Ma-Mel-86a
p53: mt (R248W)
BRAF: V600E
Ma-Mel-86c
H1299
DMSO
HCl
PLX 2µM
Daca 10µM
Doxo 1.5µM
p53β
Δ40p53α
Δ133p53α
KJC-12
α-actinin
E
p53: mt (R248W)
BRAF: V600E
Ma-Mel-86a
DMSO
0.5µM PLX
1µM PLX
KJC-12
pERK
α-actinin
F
p53: WT
BRAF: V600E
WM278
Mock
Doxo 0.75µM
PLX 1µM
PLX 2µM
p53β
KJC-12
DO-1
α-actinin
G
p53: WT
BRAF: V600E
A375M
p53: WT
BRAF: V600E
WM793B
DMSO
0.75µM Doxo
0.5µM PLX
1µM PLX
50ng/ml EGF
DMSO
0.75µM Doxo
0.5µM PLX
1µM PLX
2µM PLX
5µM PLX
Δ40p53α
p53β
KJC-12
pERK
α-actinin

H

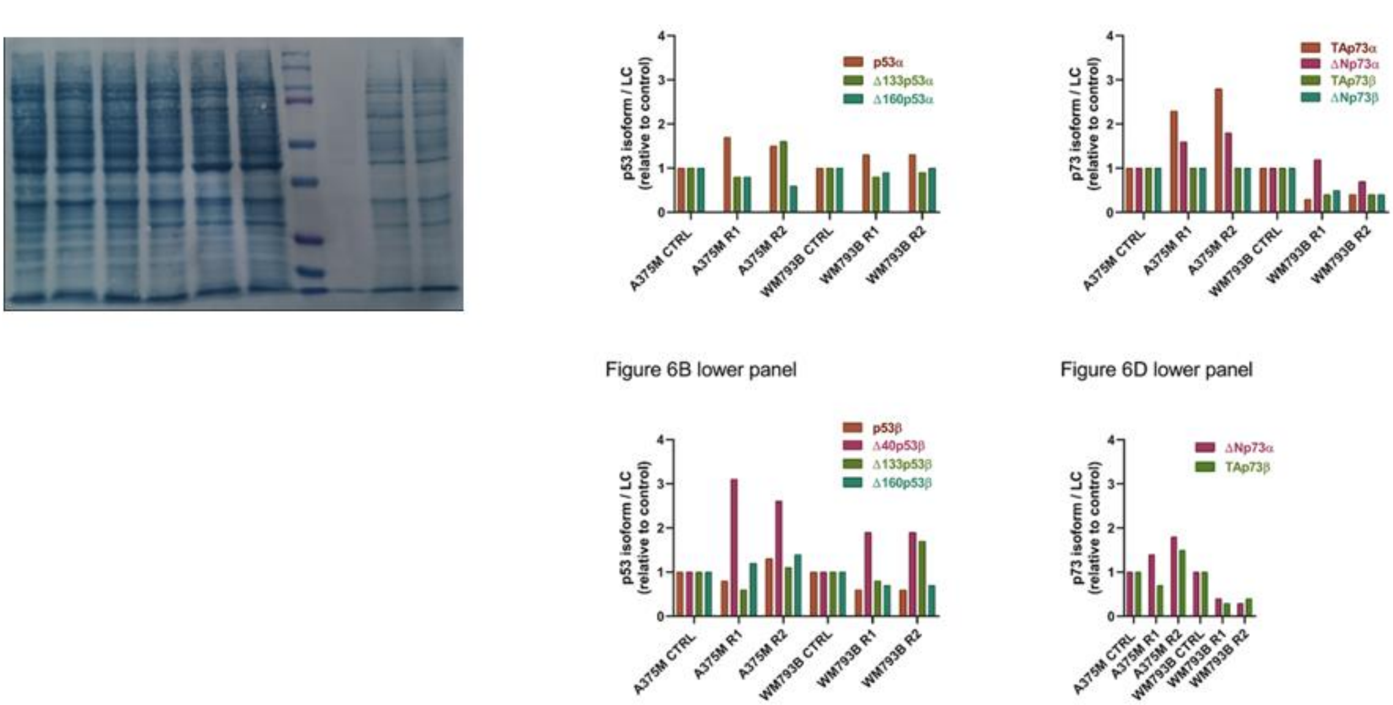

Figure S6. Analysis of the effect of different anti-cancer agents on p53 isoforms' expression. Several melanoma-derived cell lines with different status of *TP53* were treated with doxorubicin, dacarbazine, and vemurafenib (PLX-4032) anti-cancer agents at the indicated doses for 24 h. DMSO was used as control for vemurafenib and doxorubicin, while HCl for dacarbazine. (**A-G)** Endogenous p53 isoforms expression was determined by western blotting using the p53 pantropic antibody KJC-12. α-actinin was used as loading control. To better identify the different p53 isoforms, transient transfections in the p53 null cells H1299 with single isoforms were included (asterisks indicated the position on the membrane of the over-expressed p53 isoform). (**E, G)** The detection of phosphorylated form of ERK was included as a control for the sensitivity to vemurafenib in BRAF mutated cell lines Ma-Mel-86a, A375M, and WM793B. (**F)** To better evaluate the accumulation of full length p53 isoforms in response to doxorubicin and vemurafenib in WM278 cells (p53 wild-type and BRAF mutant) cell extracts were incubated with DO-1 antibody able to recognize only p53α, β, and γ isoforms. (**G)** The treatment with 50 ng/ml EGF was included as a positive control for the detection of phospho-ERK protein. (**H**) Naphthol blue stained membrane used for quantification in Figure 6B (upper panel) and quantification of the expression of p53/p73 proteins shown in Figure 6B and D using Image Lab software 6. The expression of p53/p73 proteins was normalized to the expression level of β-actin of the same samples.

Original uncropped blots

Figure 2A

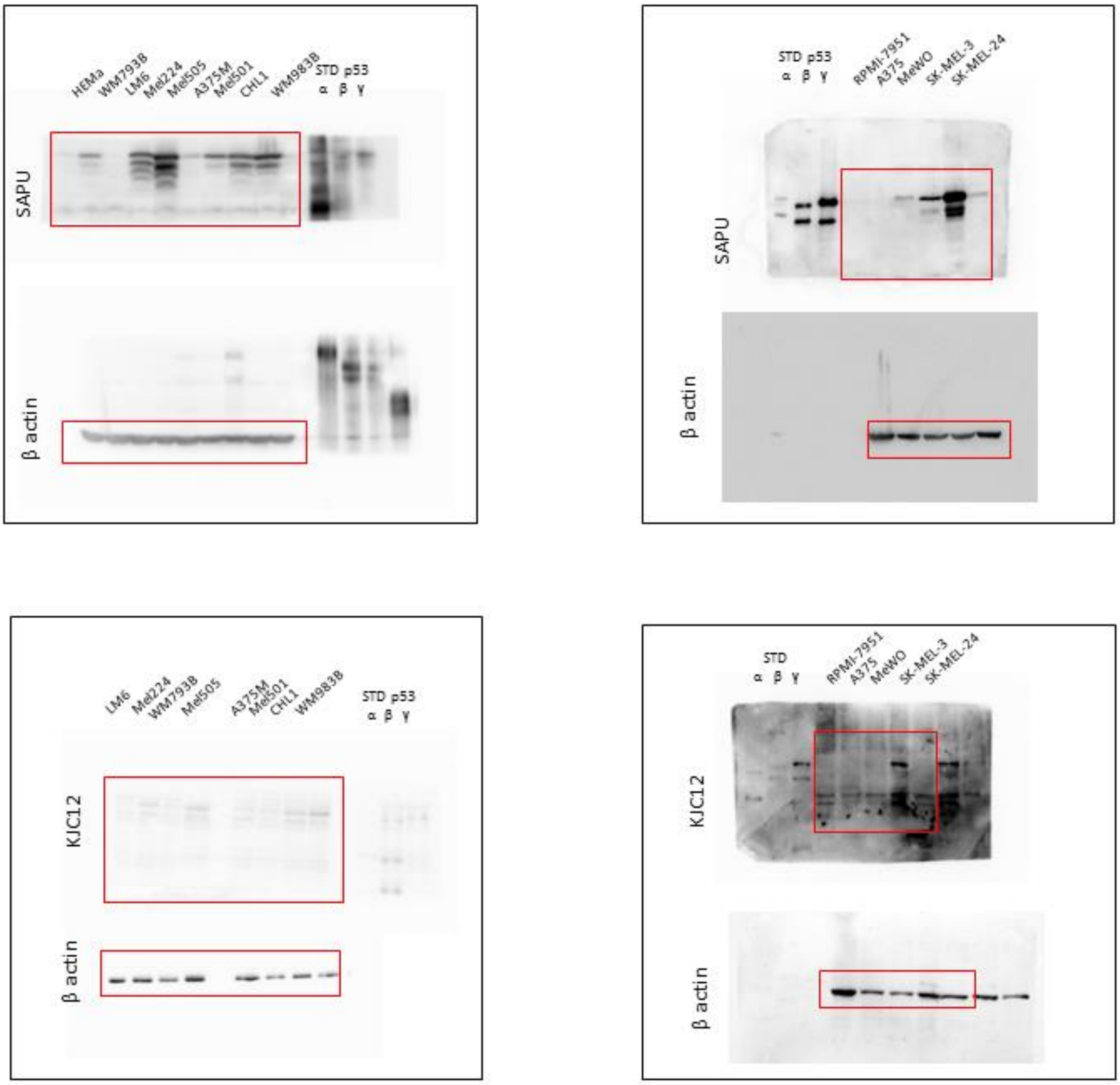

Figure 2B

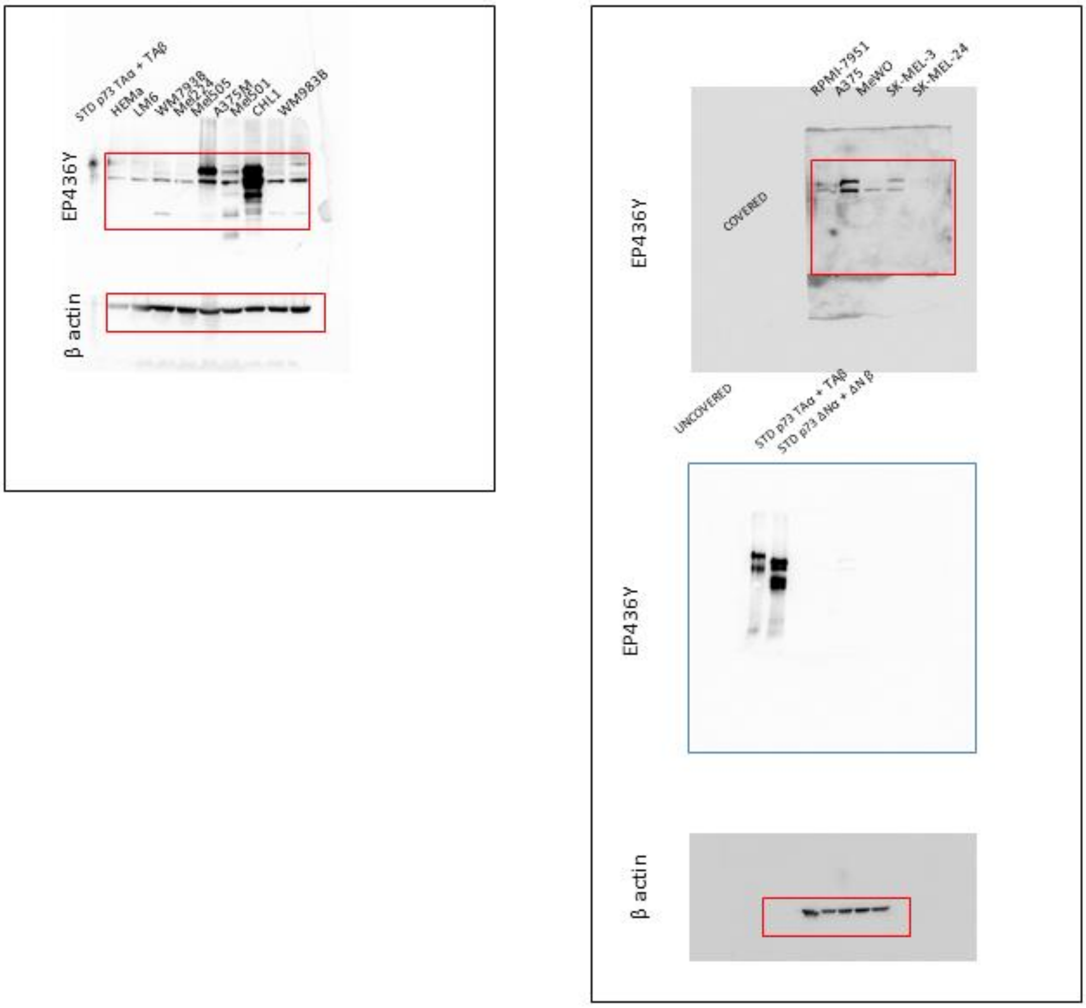

Figure 3A-B

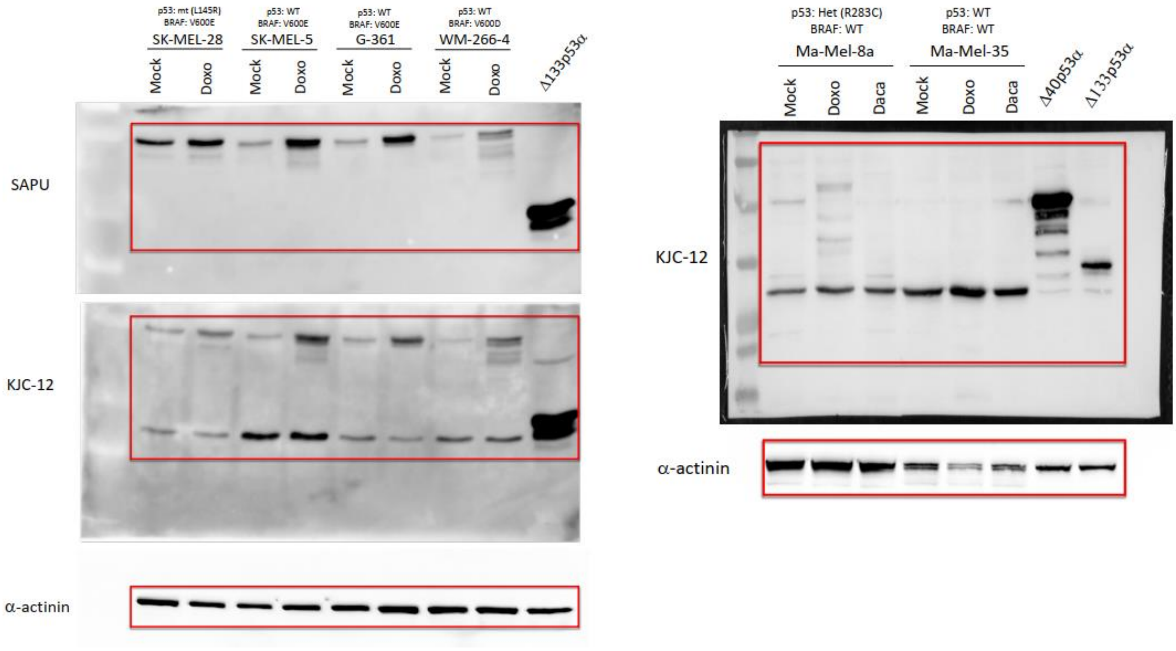

Figure 3C

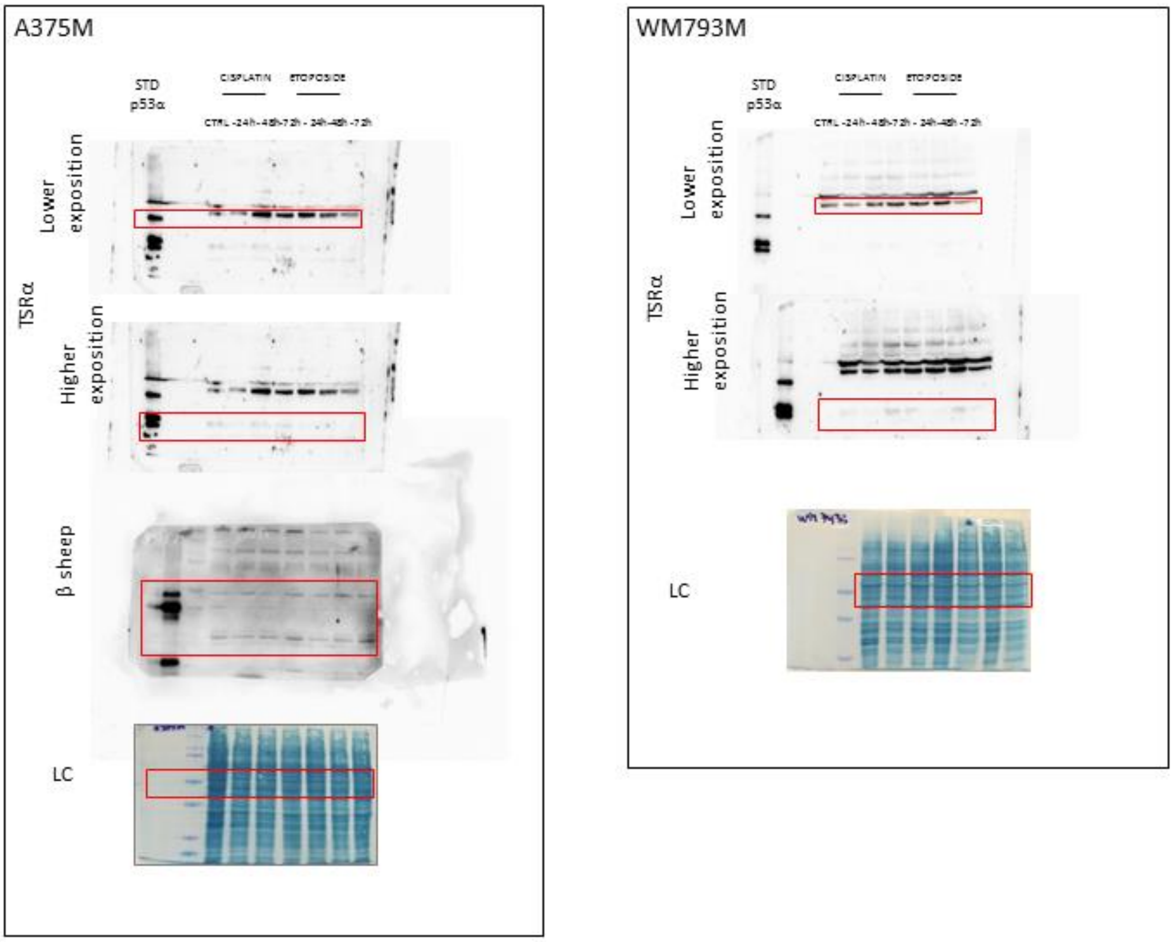

Figure 3D

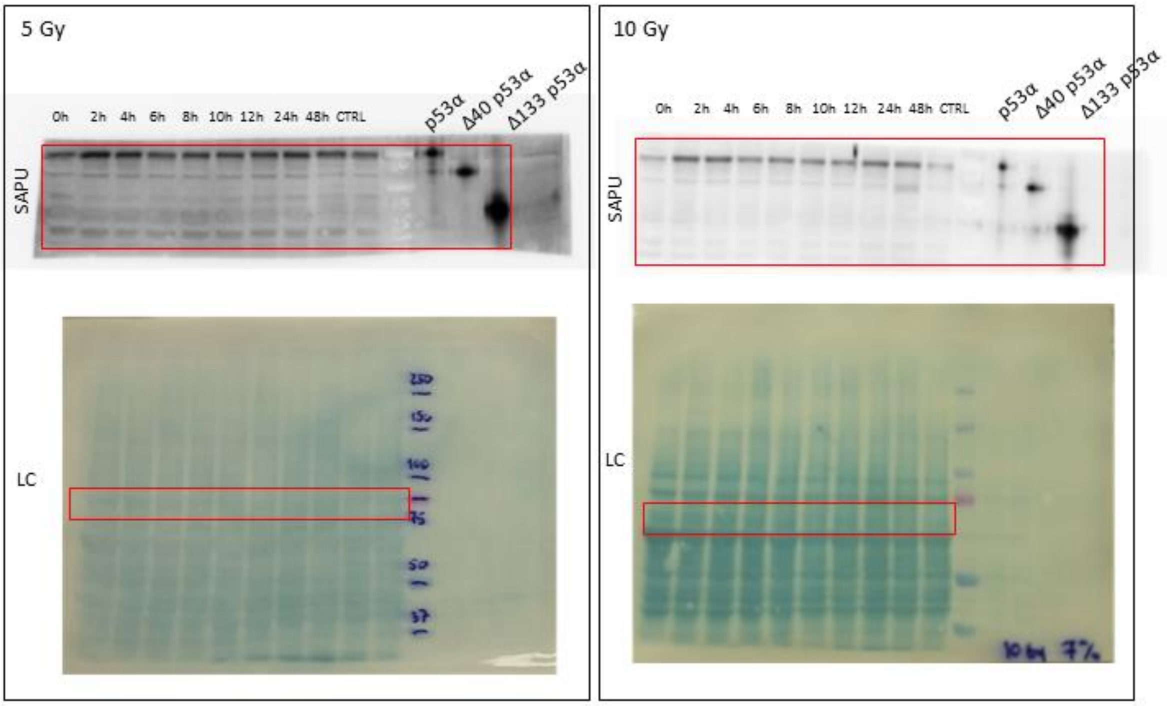

Figure 4

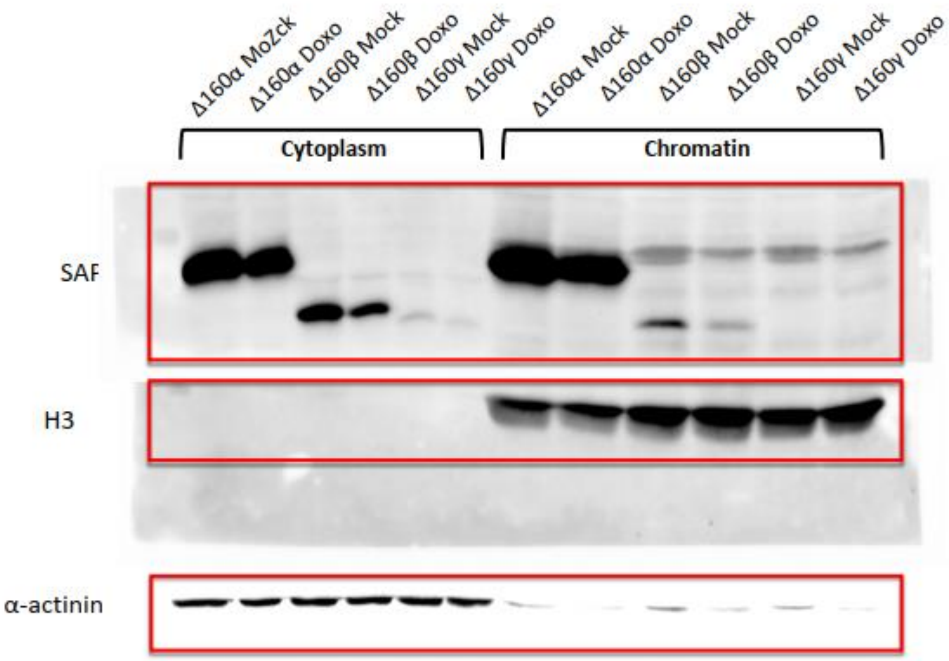

Figure 5B

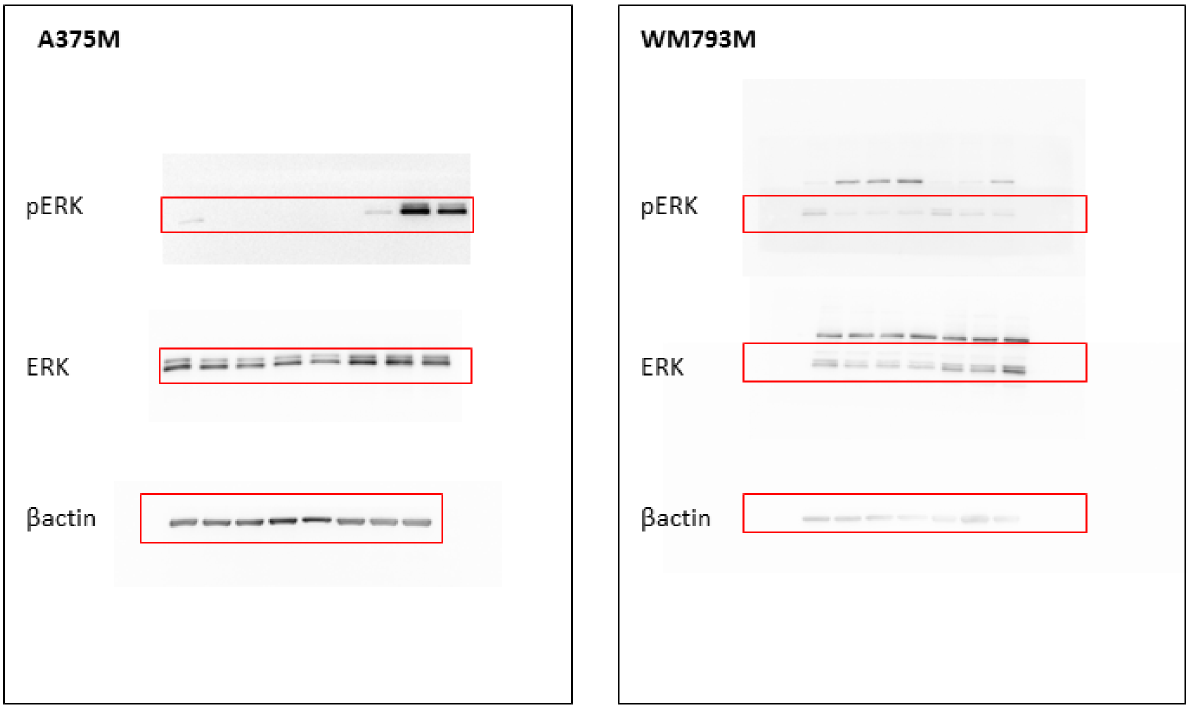

Figure 6B

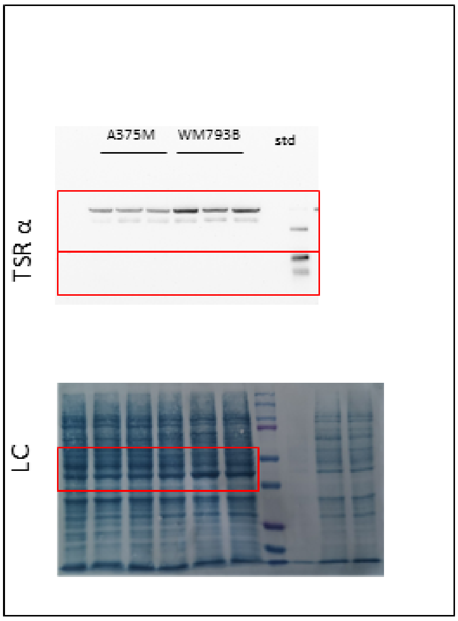

TSR α
A375M
WM793B
std
LC

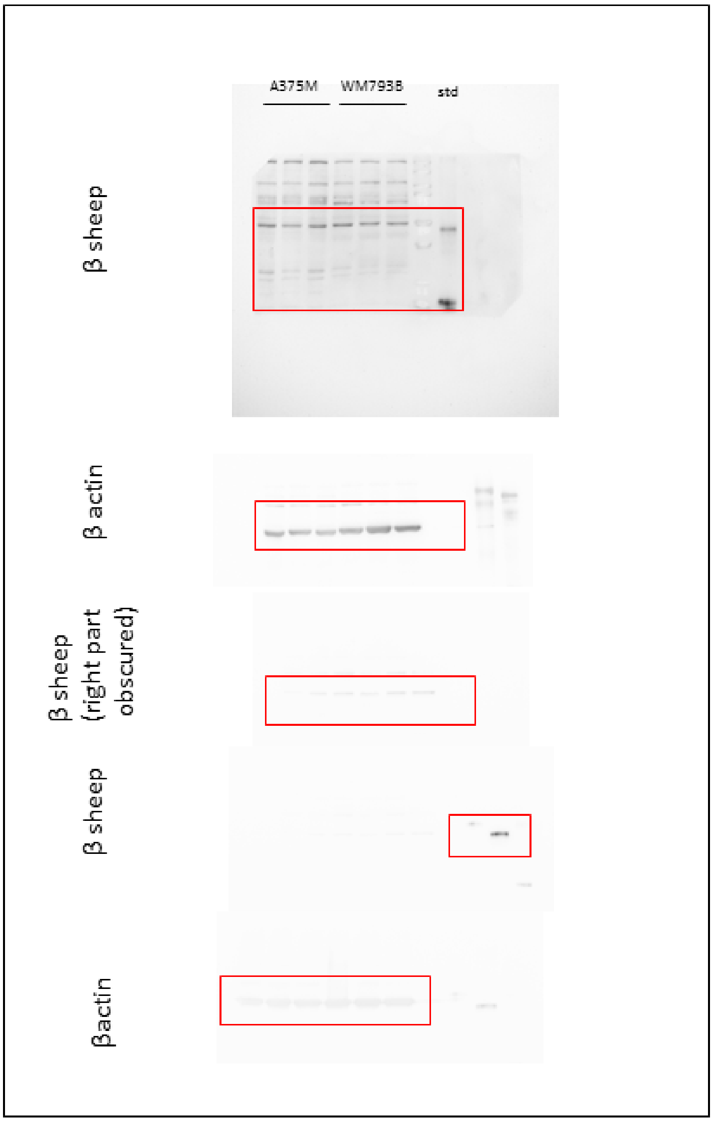

β sheep
A375M
WM793B
std
β actin
β sheep (right part obscured)
β sheep
βactin

Figure 6D

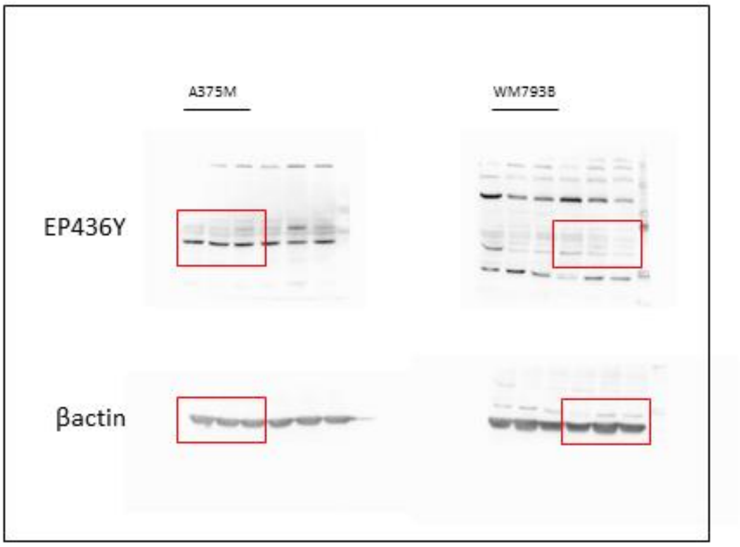

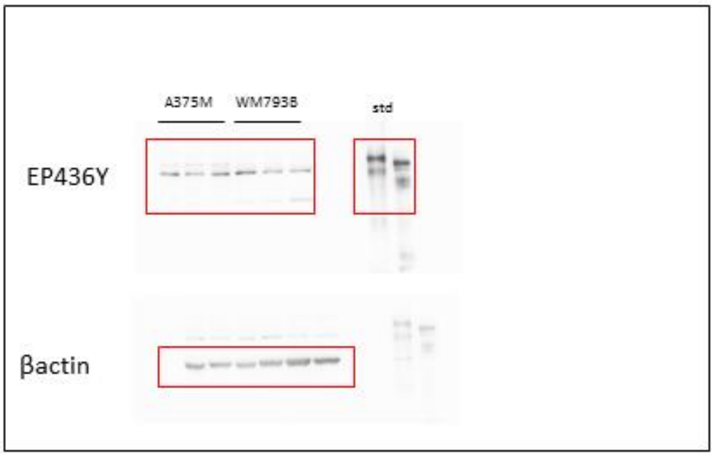

Figure S3A-D

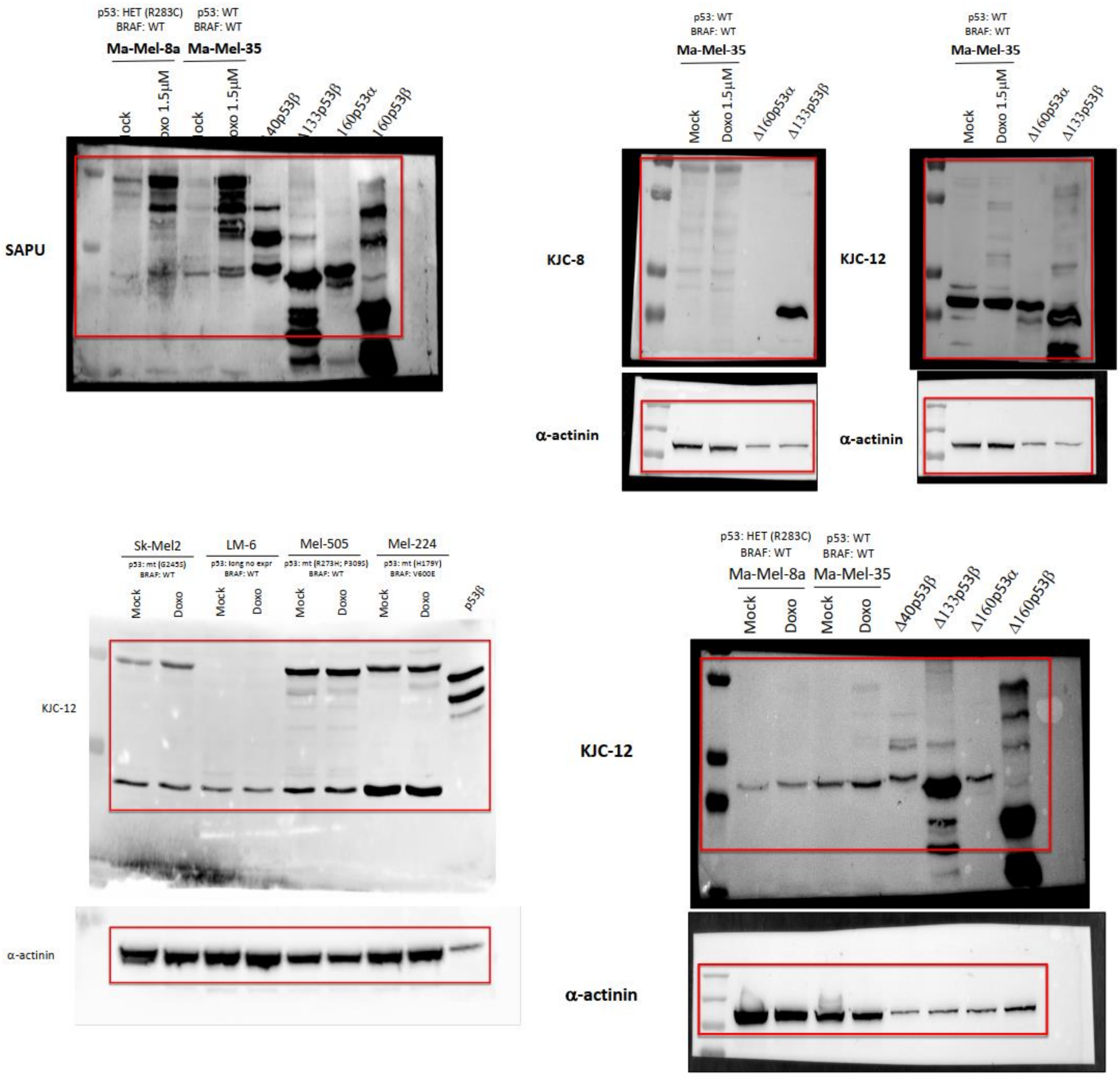
p53: HET (R283C)
BRAF: WT
Ma-Mel-8a
p53: WT
BRAF: WT
Ma-Mel-35
SAPU
Ma-Mel-35
Mock
Doxo 1.5µM
Δ160p53α
Δ133p53β
KJC-8
KJC-12
α-actinin
Sk-Mel2
LM-6
Mel-505
Mel-224
Mock
Doxo
p53β
KJC-12
α-actinin
p53: HET (R283C)
BRAF: WT
Ma-Mel-8a
p53: WT
BRAF: WT
Ma-Mel-35
Mock
Doxo
Δ40p53β
Δ133p53β
Δ160p53α
Δ160p53β
KJC-12
α-actinin

Figure S3

E

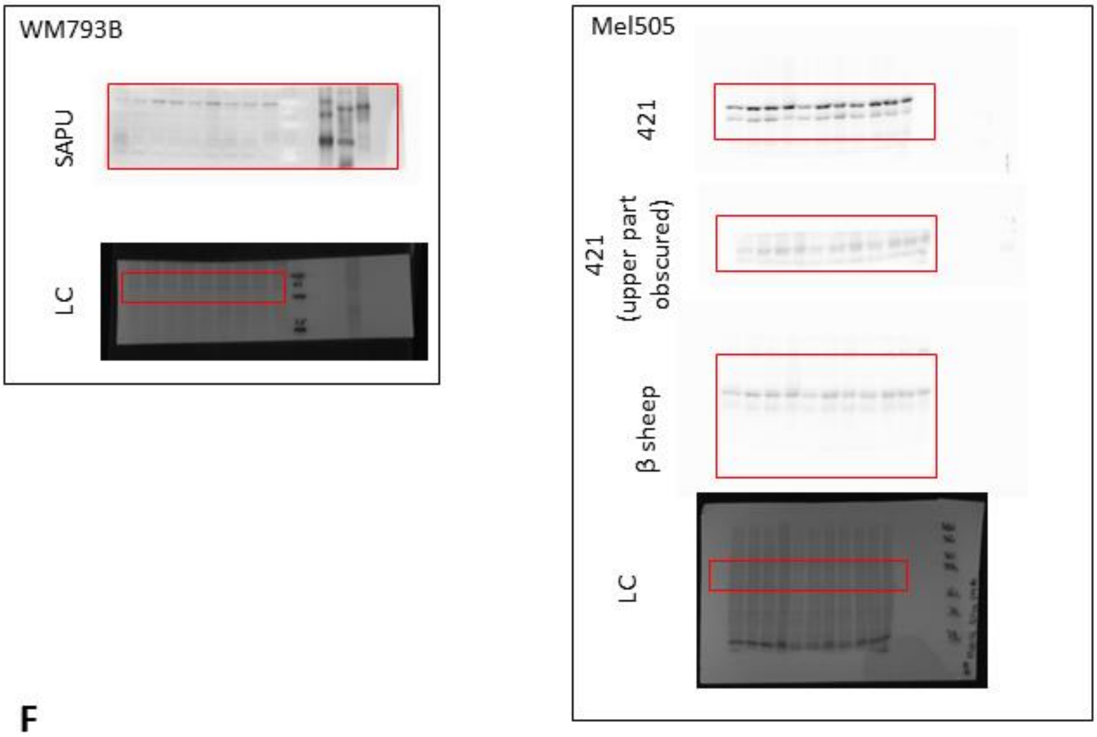
WM793B
SAPU
LC
Mel505
421
421
(upper part
obscured)
β sheep
LC

F

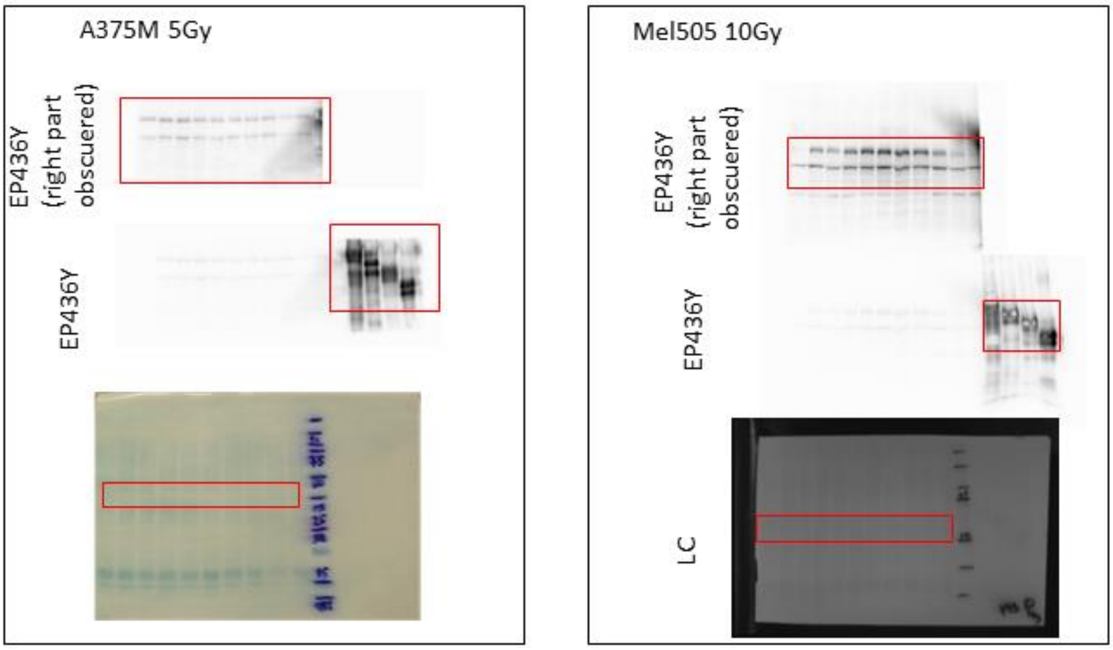
A375M 5Gy
EP436Y
(right part
obscuered)
EP436Y
Mel505 10Gy
EP436Y
(right part
obscuered)
EP436Y
LC

Figure S4

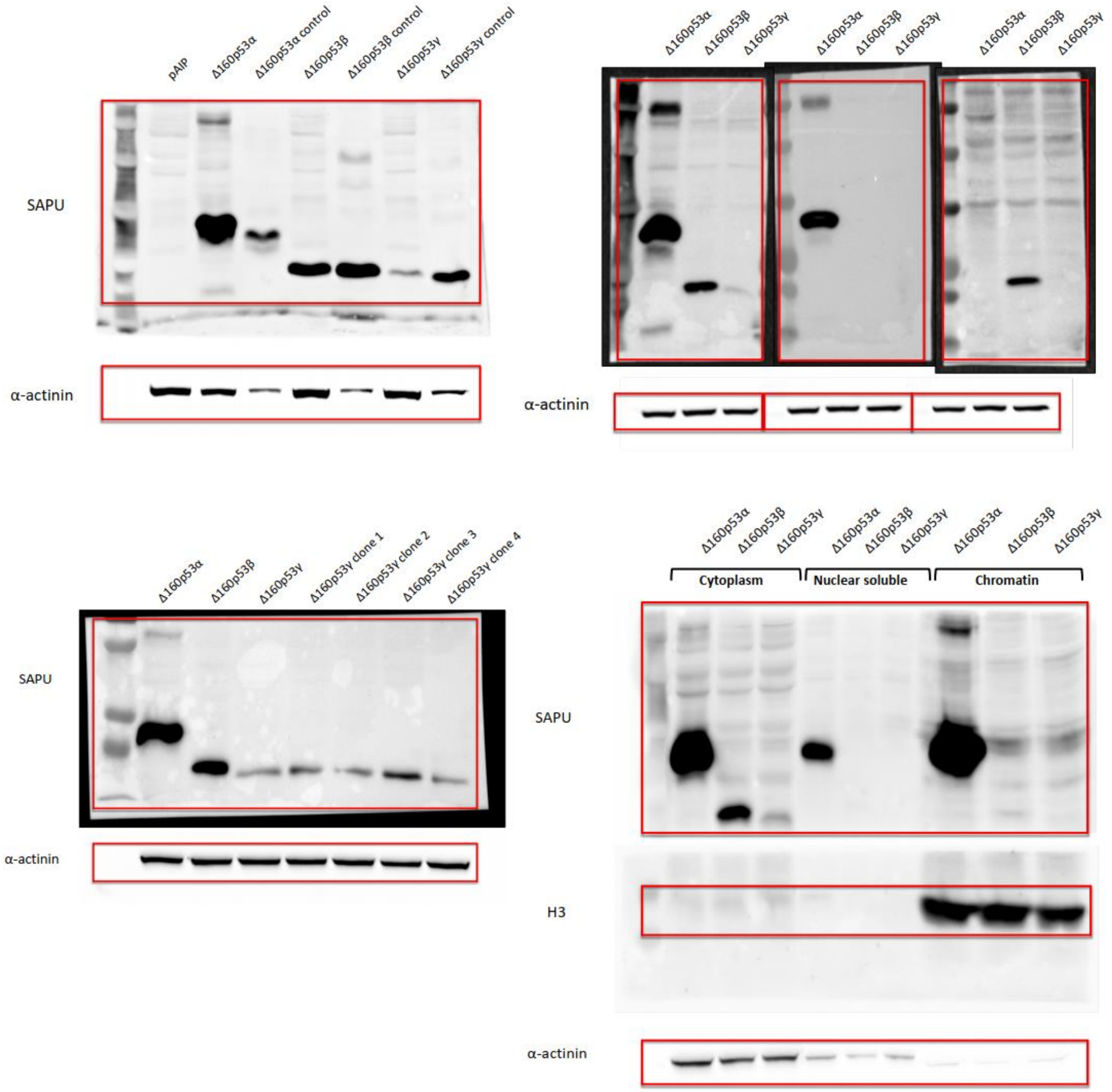
pAIP
Δ160p53α
Δ160p53α control
Δ160p53β
Δ160p53β control
Δ160p53γ
Δ160p53γ control
SAPU
α-actinin
Δ160p53α
Δ160p53β
Δ160p53γ
Δ160p53α
Δ160p53β
Δ160p53γ
Δ160p53α
Δ160p53β
Δ160p53γ
α-actinin
Δ160p53α
Δ160p53β
Δ160p53γ
Δ160p53γ clone 1
Δ160p53γ clone 2
Δ160p53γ clone 3
Δ160p53γ clone 4
SAPU
α-actinin
Δ160p53α
Δ160p53β
Δ160p53γ
Δ160p53α
Δ160p53β
Δ160p53γ
Δ160p53α
Δ160p53β
Δ160p53γ
Cytoplasm
Nuclear soluble
Chromatin
SAPU
H3
α-actinin

Figure S5A-E

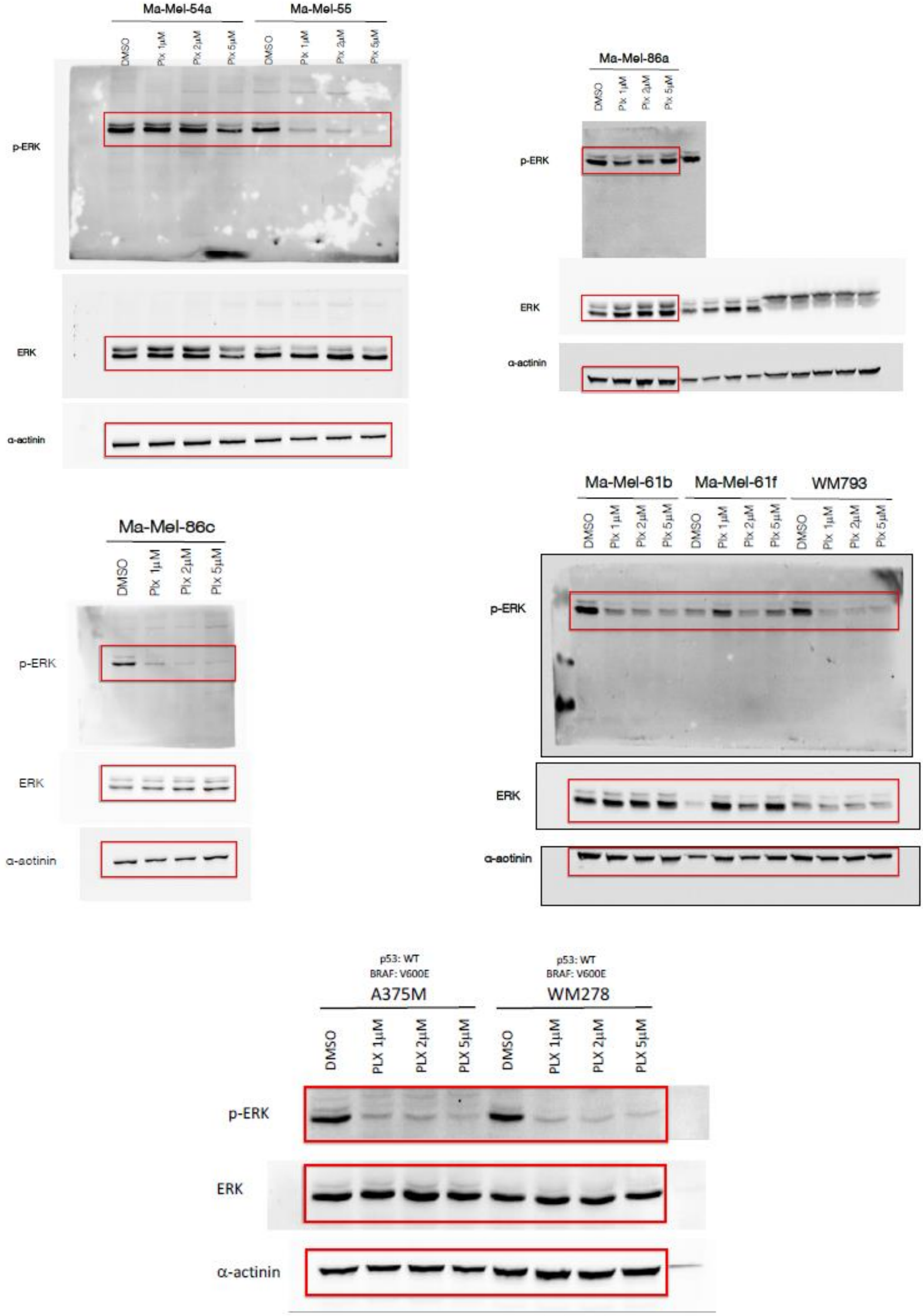

Ma-Mel-54a
Ma-Mel-55
DMSO
Plx 1μM
Plx 2μM
Plx 5μM
p-ERK
ERK
α-actinin
Ma-Mel-86a
Ma-Mel-86c
Ma-Mel-61b
Ma-Mel-61f
WM793
p53: WT
BRAF: V600E
A375M
WM278
PLX 1μM
PLX 2μM
PLX 5μM

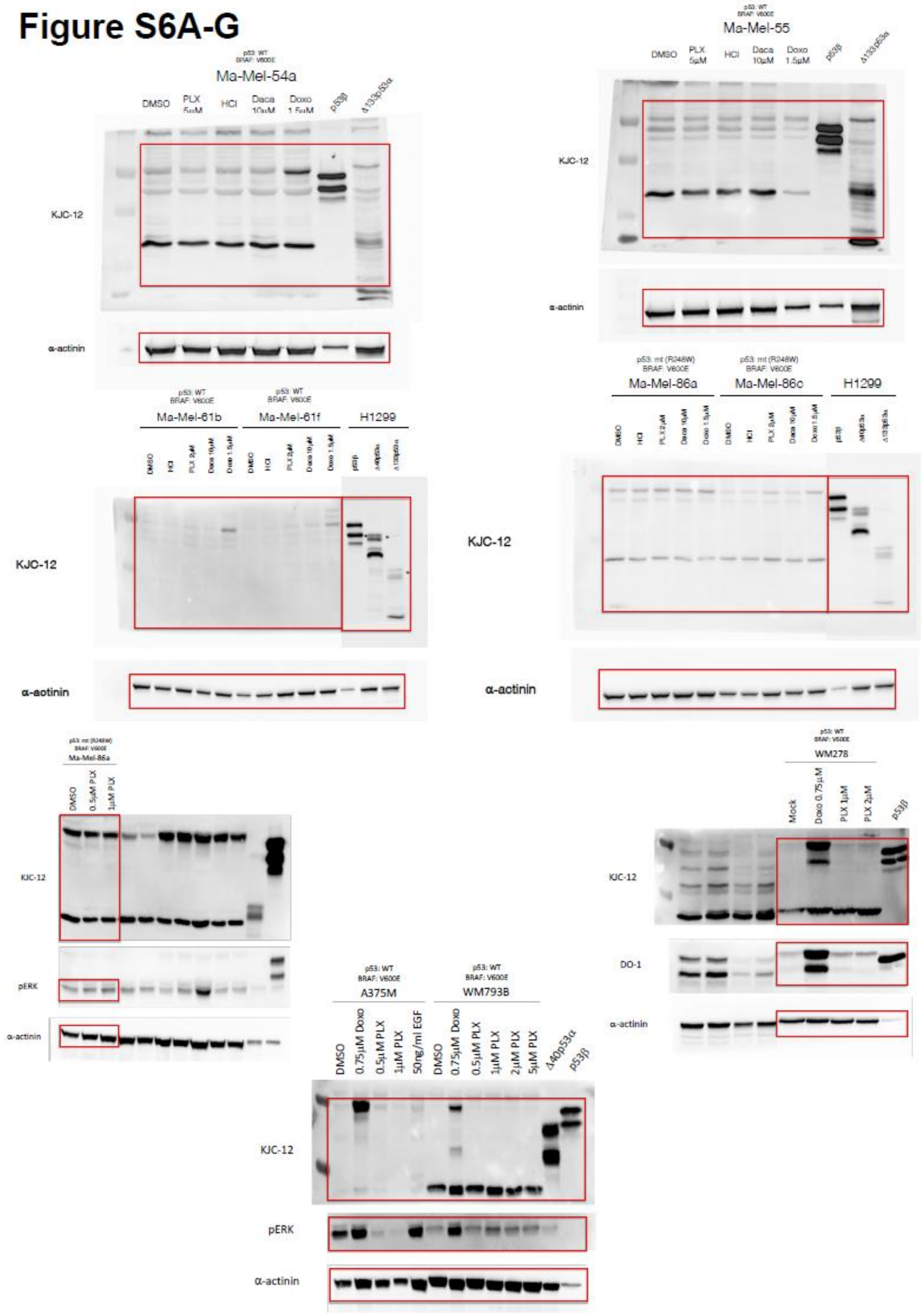

Figure S7. Original uncropped blots.
